# Plasmon‐Driven Reorientation of Interfacial Water for Wastewater Electrolysis with Light‐Emitting Diode Illumination

**DOI:** 10.1002/advs.202507147

**Published:** 2025-06-25

**Authors:** Nur Aqlili Riana Che Mohamad, Kyunghee Chae, Qiang Zhou, Wen‐Tse Huang, Hyun Jeong Lee, Jaehyun Son, Jooho Moon, Yunfei Bu, Feng Gong, Ru‐Shi Liu, Jeongwon Kim, Dong Ha Kim

**Affiliations:** ^1^ Department of Chemistry and Nanoscience Ewha Womans University Seoul 03760 Republic of Korea; ^2^ School of Engineering The University of Tokyo 7‐3‐1 Hongo, Bunkyo‐ku Tokyo 113‐8656 Japan; ^3^ Key Laboratory of Energy Thermal Conversion and Control of Ministry of Education School of Energy and Environment Southeast University Nanjing Jiangsu 211189 China; ^4^ Department of Chemistry National Taiwan University 106 Taipei Taiwan; ^5^ Department of Materials Science and Engineering Yonsei University 50 Yonsei‐ro Seodaemun‐gu Seoul 03722 Republic of Korea; ^6^ UNIST‐NUIST Research Center of Environment and Energy (UNNU) School of Environment Science and Technology Nanjing University of Information Science and Technology Nanjing 210044 P. R. China; ^7^ Nanobio·Energy Materials Center (National Research Facilities and Equipment Center) Ewha Womans University Seoul 03760 Republic of Korea; ^8^ Department of Chemistry Northwestern University Evanston IL 60208 USA; ^9^ College of Medicine Ewha Womans University 25, Magokdong‐ro 2‐gil, Gangseo‐gu Seoul 07804 Republic of Korea; ^10^ Graduate Program in Innovative Biomaterials Convergence Ewha Womans University 52 Ewhayeodae‐gil, Seodaemun‐gu Seoul 03760 Republic of Korea; ^11^ Basic Sciences Research Institute (Priority Research Institute) Ewha Womans University Seoul 03760 Republic of Korea

**Keywords:** ammonia oxidation reaction, LED illumination, plasmonic, single atom, symmetric electrolyzer

## Abstract

Regulating the orientation and dynamics of interfacial water is essential for optimizing electrocatalytic reactions, yet it remains challenging due to its intrinsic disorder. Here, it is demonstrated that localized surface plasmon resonance (LSPR) on an Ir single‐atom Au catalyst actively restructures the hydrogen‐bond (HB) network, accelerating ammonia oxidation reaction kinetics. In situ Raman spectroscopy and density functional theory calculations reveal that plasmonic excitation shifts the HB network toward a more flexible configuration, favoring the formation of three‐coordinated hydrogen‐bonded water (3HB·H_2_O) while suppressing cation‐associated species (K^+^·H_2_O). This transformation enhances the dehydrogenation process and stabilizes reaction intermediates, leading to a 28% increase in NH_3_ oxidation kinetics. *Operando* X‐ray absorption spectroscopy further confirms that LSPR‐driven polarization at the Ir active site compresses the Ir─O bond from 1.73 to ≈1.58 Å by generating electron vacancies, thereby accelerating deprotonation with ^*^OH during oxidative electrolysis. Extending this principle to an LED‐driven plasmon‐assisted symmetric wastewater electrolyzer, achieving a 40‐fold current enhancement and 94% ammonia removal efficiency over 120 h at 1 V under landfill leachate‐like conditions.

## Graphical abstract

Localized surface plasmon resonance (LSPR) on Ir single‐atom Au catalysts restructures the hydrogen‐bond network, promoting flexible 3HB·H_2_O formation while suppressing K^+^·H_2_O species. This modulation of interfacial water enhances dehydrogenation and stabilizes reaction intermediates, leading to an increase in ammonia oxidation kinetics.

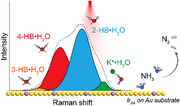

## 1 Introduction

Interfacial water is a critical mediator in electrochemical reactions, governing key interfacial interactions and significantly influencing reaction pathways, kinetics, and overall efficiency.^[^ ^1^, ^2^, ^3^ ^]^ The hydrogen‐bonding (HB) networks within this layer shape catalyst performance by modulating electric field distributions, adsorption behavior, and intermediate stabilization.^[^ ^4^ ^]^ These interactions dictate the adsorption distance and binding energies of intermediates at active sites, thereby controlling reaction selectivity and efficiency. Strategic tuning of interfacial water properties thus provides a convincing avenue for optimizing electrochemical processes in energy conversion and storage. Building on this foundation, plasmon‐mediated reactions leverage nanoscale phenomena to enhance catalytic efficiency under mild conditions.^[^ ^5^, ^6^, ^7^, ^8^ ^]^ Localized surface plasmon resonance (LSPR), in particular, alters surface dynamics by driving adsorption and desorption behaviors of molecular and ionic intermediates.^[^ ^9^, ^10^, ^11^, ^12^, ^13^, ^14^ ^]^ This phenomenon arises through the collective excitation of hot carriers (electrons and holes) on plasmonic metal surfaces, alongside the generation of intense, spatially localized oscillating electric fields. These effects induce localized electronic and structural perturbations, driving oxidation state changes near nanoparticles and opening new electrochemical pathways with enhanced catalytic precision and efficiency.^[^ ^15^, ^16^ ^]^

**Figure 1:**
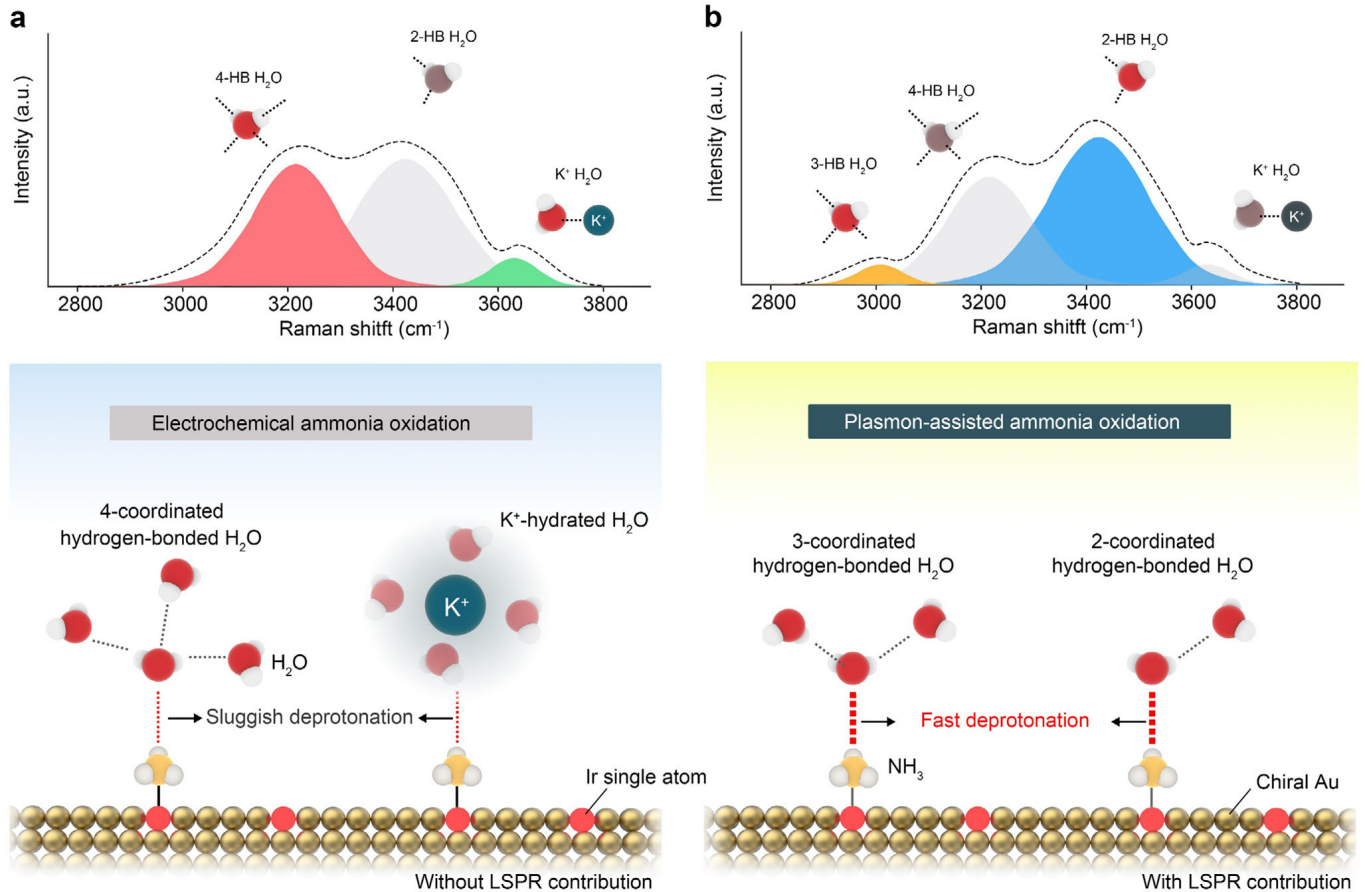
Schematic description of interfacial water configurations in plasmon‐driven AOR process on Ir_SA_Au‐L electrocatalyst. a) Under non‐plasmonic excitation, the O–H stretching region of the Raman spectra measured on a surface‐enhanced Raman spectroscopy (SERS)‐active substrate is deconvoluted into four subbands located at ≈3200, ≈3400, and ≈3600 cm^−1^. These correspond to water molecules with four hydrogen bonds (4‐HB·H_2_O), two hydrogen bonds (2‐HB·H_2_O), and hydrated cations (K^+^·H_2_O), respectively. b) Upon plasmonic excitation, localized surface plasmon resonance (LSPR) induces dynamic restructuring of the interfacial water network, promoting the formation of 3‐HB·H_2_O and increasing the proportion of 2‐HB·H_2_O species. This restructuring facilitates rapid deprotonation, thereby accelerating the ammonia oxidation process.

Harnessing this concept, we demonstrate how LSPR dynamically modulates interfacial water, unlocking new catalytic possibilities for ammonia electrolysis. The sluggish kinetics of the ammonia oxidation reaction (AOR) pose a significant challenge due to its intricate six‐electron transfer process, requiring efficient coupling between NH_3_ species and OH^–^ adsorbates.^[^ ^17^, ^18^ ^]^ To overcome these limitations, we designed an Ir single‐atom catalyst on a chiral Au substrate (Ir_SA_Au‐L), leveraging both the high efficacy of single atom catalysts and plasmonic effects to optimize hydrogen‐bond (HB) networks and accelerate AOR kinetics while maintaining long‐term operational stability (**Figure** **1**). The chiral Au substrate serves a dual function: it selectively interacts with amine and thiol groups (e.g., L‐cysteine) to direct the controlled nucleation of Au nanoparticles while ensuring the uniform dispersion of Ir single atoms. This precise atomic arrangement optimizes active site accessibility, creating an ideal platform for plasmon‐driven catalytic enhancement.^[^ ^19^, ^20^ ^]^ To optimize strong plasmon‐water coupling and ensure uniform Ir single atom dispersion, Ir_SA_Au‐L was selected as the representative system as the racemic Ir_SA_Au‐L/D exhibited limited enhancement and lower AOR performance under illumination. In situ Raman spectroscopy further reveals that plasmonic excitation disrupts rigid tetrahedral 4‐HB configurations, transforming them into more flexible 3‐ and 2‐HB structures. This restructuring enhances the stabilization of key intermediates while increasing reactant‐water collision frequencies, both of which are critical for efficient NH_3_ oxidation. Furthermore, plasmon‐induced suppression of hydrated cations minimizes competitive hydrogen adsorption, creating a more favorable environment for NH_3_ dehydrogenation process. Density functional theory (DFT) calculations support these findings, illustrating how the redistribution of electronic states under plasmonic conditions strengthens intermediate interactions and accelerates reaction kinetics. Beyond water structuring, *operando* X‐ray absorption spectroscopy (XAS) reveals that LSPR‐driven polarization at the single‐atom Ir site compresses the Ir─O bond, a key electronic reconfiguration that facilitates electron transfer and stabilizes oxygen intermediates essential for sustaining high catalytic activity. Extending this approach to a light‐emitting diode (LED)‐driven plasmon‐assisted wastewater electrolyzer, we achieve a 40‐fold increase in current and 94% NH_3_ removal efficiency over 120 h at 1 V under landfill leachate‐like wastewater conditions. These findings underscore the potential of plasmonic single‐atom catalysts to transform ammonia electrolysis, paving the way for more efficient and scalable nitrogen waste remediation technologies.

## 2 Results and Discussion

### 2.1 Materials Characterization

**Figure 2:**
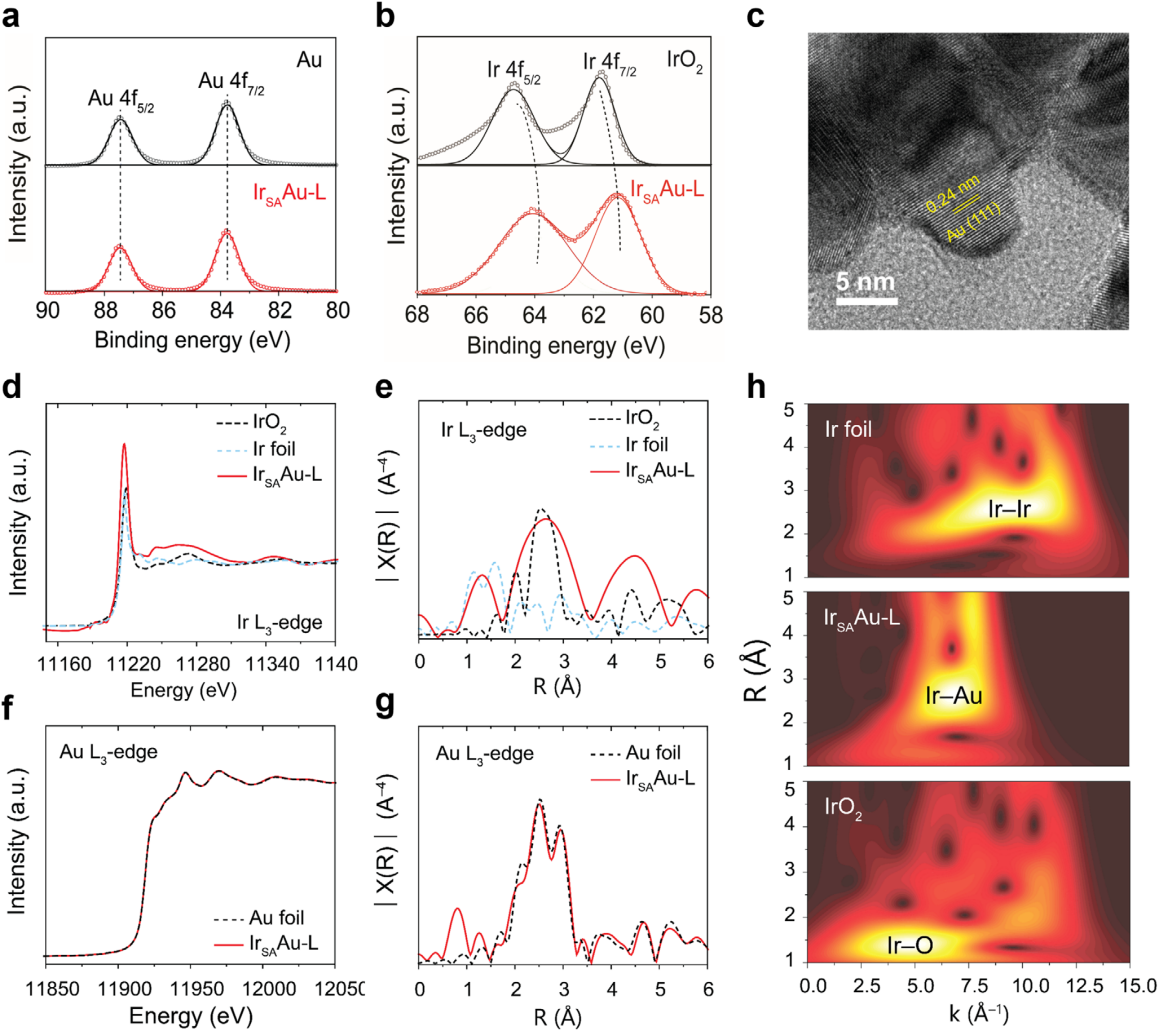
Structural characterizations and X‐ray absorption spectroscopy. a) Au 4f XPS spectra of chiral Au and Ir_SA_Au‐L. b) Ir 4f XPS spectra of IrO_2_ and Ir_SA_Au‐L. c) TEM images Au‐L. d,e) Ir *L_3_*‐edge XANES spectra, and corresponding *k^2^*‐weighted Fourier transform (FT) of Ir *L_3_*‐edge EXAFS signal for Ir_SA_Au‐L, Ir foil, and IrO_2_. f,g) Au *L_3_*‐edge XANES spectra with standard Au foil and Ir_SA_Au‐L, and corresponding FT *k^2^*‐weighted Au *L_3_*‐edge EXAFS in R space. h) Wavelet transform spectra for Ir foil, IrO_2_, and Ir_SA_Au‐L.

A facile two‐step electrodeposition method was employed to deposit Ir single atom (SA) on a chiral Au substrate, forming an Ir_SA_Au‐L electrocatalyst. X‐ray diffraction (XRD) (Figure S1, Supporting Information) verified the high crystallinity of the Ir_SA_Au‐L samples, with no detectable Ir peaks due to the minimal Ir loading (1.4 wt.%), as measured by inductively coupled plasma‐mass spectroscopy (ICP‐MS). X‐ray photoelectron spectroscopy (XPS) analysis (**Figure** **2**a,b) of the Ir 4f region showed doublets at 61.1 eV (Ir 4f_7/2_) and 63.8 eV (Ir 4f_5/2_), shifted to lower binding energies compared to IrO_2_ (61.7 and 64.6 eV, respectively). This shift indicates electron transfer from Ir to Au, indicating strong electronic interactions in the bimetallic Ir–Au system, consistent with previous findings.^[^ ^21^, ^22^ ^]^ TEM analysis revealed that the incorporation of Ir single atoms preserved the chiral phase structure of the Au nanoflowers (NFs), as evidenced by the well‐defined Au(111) lattice fringes observed across multiple domains (Figure 2c). Elemental mapping by EDS confirmed the homogeneous spatial distribution of both Au and Ir (Figures S2 and S3, Supporting Information), indicating successful atomic‐level integration. High‐angle annular dark‐field scanning transmission electron microscopy (HAADF‐STEM) imaging (Figure S4, Supporting Information) provided additional evidence for the uniform dispersion of Ir single atoms on the Au substrate. High‐resolution images revealed well‐isolated Ir atoms, corroborated by intensity line profiles extracted from selected regions in the HAADF‐STEM image. These profiles exhibited distinct atomic contrast variations, consistent with the presence of individual Ir atoms embedded within the Au lattice.

The atomic and electronic structures of single Ir atoms on chiral Au‐L (Ir_SA_Au‐L) nanoparticles were systematically analyzed using X‐ray absorption fine structure spectroscopy (XAFS). Figure 2d shows the X‐ray absorption near‐edge structure (XANES) spectra at the Ir *L_3_*‐edge to closely monitor the state and atomic configuration of Ir_SA_Au‐L. The XANES profile of Ir_SA_Au‐L closely resembles that of metallic Ir, indicating that most Ir atoms exist predominantly in a metallic state.^[^ ^23^ ^]^ The white line in the Ir_SA_Au‐L spectrum shifts by +0.5 eV relative to Ir foil (0) and by −0.9 eV compared to IrO_2_ (+4), indicating distinct electronic interactions. The white line intensity, which correlates with the density of empty d orbital states, follows the trend: Ir_SA_Au‐L > IrO_2_ > Ir foil. This suggests the presence of unsaturated Ir coordination sites or IrO_x_ species.^[^ ^24^ ^]^ The increased density of empty *d* orbital states in Ir_SA_Au‐L is expected to enhance its electrocatalytic performance.^[^ ^23^, ^25^ ^]^ EXAFS analysis at the Ir *L_3_*‐edge reveals two distinct peaks in R space (Figure 2e), identifying the first coordination shell of Ir in Ir_SA_Au‐L as Ir─O and Ir─Au bonds at distances of 1.07 and 2.18 Å, respectively, highlighting the stable structural configuration of Ir_SA_Au‐L. Figure 2f shows the Au *L_3_*‐edge XANES spectra of Ir_SA_Au‐L and standard Au foil. A clear distinction in the spectra is difficult to discern due to the fully populated *5d* orbital of Au metal, however, a slight leftward shift in the XANES edge jump suggests potential alterations in the electronic structure of Au in the vicinity of the Ir atoms.^[^ ^26^ ^]^ Meanwhile, the k^2^‐weighted Fourier‐transformed Au *L_3_*‐edge EXAFS spectra (Figure 2g) of Ir_SA_Au‐L closely resemble those of Au foil, with the peak broadening attributed to the coexistence of Au–Ir and Au–Au interactions. Notably, Ir undergoes pronounced electronic modulation, the corresponding changes in Au remain below the detection threshold of XANES, reflecting the intrinsic electronic asymmetry typical of noble metal systems and aligning with prior reports on supported single‐atom bimetallic catalysts.^[^ ^27^ ^]^

So, to depth understanding the Au configuration, we further conducted the wavelet transform from the EXAFS profile. As shown in Figure 2h, the intensity peaks for Ir and IrO_2_ occur at ≈11 and ≈6 Å^−1^, corresponding to Ir─Ir and Ir─O bonds, respectively. In contrast, Ir_SA_Au‐L shows a distinct peak near 7.5 Å^−1^, indicative of Ir−Au interactions.^[^ ^23^ ^]^ Despite the smaller distances and coordination numbers of Ir−Au in Ir_SA_Au‐L against Ir−Ir or Ir−O, the Ir−Au peak remains prominent due to differences in reciprocal *k* space.

Circular dichroism (CD) spectra further revealed distinct mirror symmetry, validating the induction of opposite chirality in the Au NFs synthesized with D‐cysteine: Au^3+^ (Au‐D NF) and L‐cysteine: Au^3+^ (Au‐L NF) (Figure S5, Supporting Information). These findings provide strong evidence for successful chirality induction. It is also noteworthy that the spectra were obtained from films prepared independently, and minor variations in film thickness or morphology could potentially influence the observed symmetry. In contrast, the use of DL‐cysteine: Au^3+^ complexes resulted in an achiral film, lacking any mirror symmetry. The spin‐dependent current of chiral Au NFs was studied using magnetic conductive atomic force microscopy (mc‐AFM) (Figure S6, Supporting Information). Ferromagnetic CoCr tips, pre‐magnetized in both northward and southward directions, were used to measure spin‐polarized currents across a voltage range from −1.5 to 1.5 V. To ensure data reliability, over 30 different points on each sample were measured. For the Ir_SA_Au‐L, the current was significantly higher under TipUp compared to TipDown, indicating a preference for up‐spin charge carriers. The Ir_SA_Au‐D, however, exhibited increased current under downward tip magnetization and the achiral Ir_SA_Au‐D/L showed no significant current variation with different tip magnetizations. The calculated spin polarization for Ir_SA_Au‐L was found to be 81.4%, indicating a pronounced spin‐selective behavior. In contrast, the Ir_SA_Au‐D/L exhibited no significant current variation​, further verifying the spin‐dependent current response is directly driven by the chirality‐induced properties of the Au.^[^ ^28^, ^29^ ^]^

### 2.2 Electrochemical Measurement and Mechanistic Analysis

**Figure 3:**
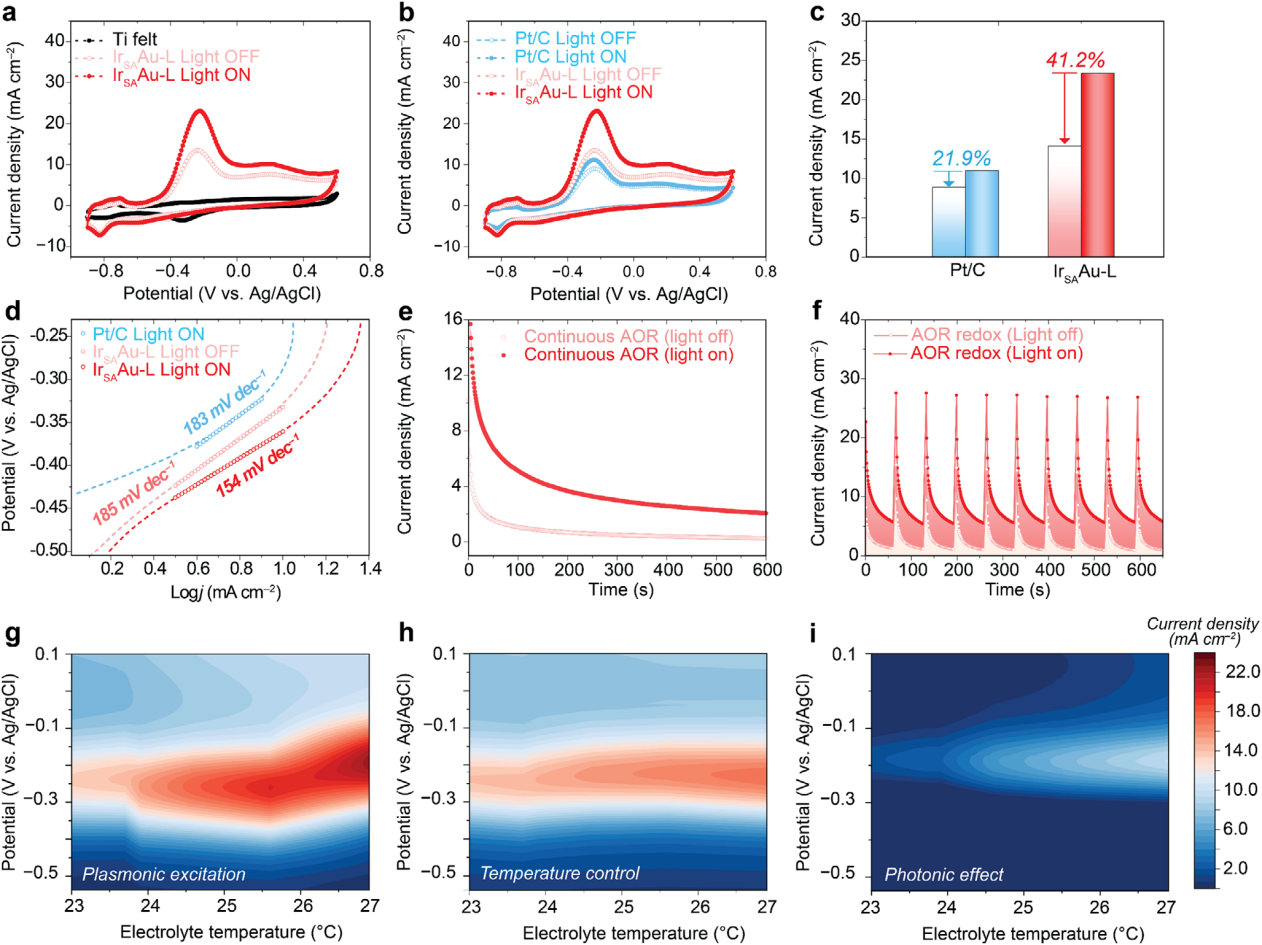
Electrochemical measurement of AOR. CV measurement at 10 mV s^−1^ of a) control Ti felt and Ir_SA_Au‐L, with and without plasmonic illumination. Comparison with b) the commercial Pt/C electrocatalyst and c) the corresponding difference in enhancement of AOR peak current density (solid bar represents reaction under illumination). d) The Tafel slope value of illuminated Pt/C and Ir_SA_Au‐L and Ir_SA_Au‐L under dark. AOR stability test measurement in continuous mode e,f) redox test of Ir_SA_Au‐L with and without plasmonic irradiation. g,h) The contour plot of AOR CV curves under different power irradiation, different electrolyte temperatures (in accordance with the applied laser power), and i) the photonic effect as a function of applied potential and electrolyte temperature. The photonic effect profile is calculated by the differences between plasmonic excitation effect and the temperature control profile.

The electrocatalytic AOR activity of the Ir_SA_Au‐L electrocatalyst was assessed in a three‐electrode setup using 0.05 m NH_4_Cl in 0.5 m KOH as electrolyte. **Figure** **3**a presents the cyclic voltammetry (CV) curves of Ir_SA_Au‐L under plasmonic irradiation, revealing a peak current intensity (22.6 mA cm^−2^) increase at −0.2 V versus Ag/AgCl, with 40.27% enhancement relative to dark conditions (13.3 mA cm^−2^). As summarized in Table S1 (Supporting Information), Ir_SA_Au‐L exhibits one of the highest AOR peak current densities reported under comparable conditions, underscoring its superior electrocatalytic performance. Similarly, lower AOR activity observed in the Ir_SA_Au‐D and racemic Ir_SA_Au‐L/D electrodes further supports the optimal configuration of the Ir_SA_Au‐L electrode (Figure S7, Supporting Information). Notably, the electrochemically active surface area (ECSA) of IrAu‐L was maximized under plasmonic irradiation, surpassing both the unilluminated and Ir‐free (Au‐L) configurations. The lower AOR activity observed in the Au‐D and racemic Au‐L/D electrodes further supports the optimal configuration of the Ir_SA_Au‐L electrode (Figure S8, Supporting Information). Moreover, the optimal deposition of Ir single atoms on the Au‐L substrate, which resulted in the highest AOR activity, suggests an additional level of interfacial control, potentially mediated by structural, electronic, or local electromagnetic field effects that modulate the hydration shell and the reaction microenvironment surrounding the Ir single‐atom sites.^[^ ^30^, ^31^, ^32^ ^]^ In comparison, commercial Pt/C showed notably lower peak intensities (Figure 3b,c) with only a 21.9% increment under irradiation, underscoring the substantial increase achieved by Ir_SA_Au‐L. The Ir_SA_Au‐L exhibited the smallest Tafel slope (154 mV dec^−1^) under irradiation, indicating superior AOR kinetics and electron transfer efficiency on the Au‐L substrate (Figure 3d). Durability tests, including chronopotentiometry at −0.2 V versus Ag/AgCl and redox AOR cycling with and without irradiation, demonstrated high stability in plasmonically‐irradiated Ir_SA_Au‐L, with negligible current decay observed (Figure 3e,f), attributed to the robust Ir single‐atom structure on the Au‐L substrate.

Further investigation into wavelength‐dependent selectivity supports the role of plasmonic effect in enhancing AOR performance. Excitation with 655 nm light, matching the LSPR of Au‐L, maximized selectivity and activity of AOR (Figure S9a, Supporting Information), with the LSPR effect contributing predominantly to these enhancements. The AOR enhancement activity under plasmonic illumination is attributed to two key mechanisms of surface plasmon relaxation, namely energetic carrier generation (photoelectronic effect) and localized photothermal heating at the interface. To disentangle these contributions, systematic evaluations of AOR responses under varying laser powers and reaction conditions were conducted (Figure 3g,h). Increasing illumination power elevates both temperature and hot‐carrier generation linearly, as previously reported.^[^ ^33^, ^34^ ^]^ Reaction kinetics and diffusion, however, follow a nonlinear dependence on temperature as described by the Arrhenius equation.^[^ ^35^ ^]^ Accordingly, the linear increase in photocurrent with illumination intensity strongly implicates hot carriers as the dominant mechanism driving catalytic enhancement.

This distinction verifies that the observed catalytic improvements primarily originate from hot‐carrier dynamics rather than thermal effects, underscoring their pivotal role in enhancing AOR efficiency. When the laser power was incrementally increased from 0.3 to 1.5 W, the AOR peak current density demonstrated a linear rise from 14.2 to 22.6 mA cm^−2^ (Figure 3g; Figure S9a, Supporting Information), substantiating the contribution of hot carriers to the AOR catalytic process.^[^ ^36^, ^37^ ^]^ This is consistent with methods distinguishing hot‐carrier contributions from thermal effects, as shown by the linear increase in photocurrent under rising illumination, contrasting the nonlinear response typical of thermal‐driven kinetics.^[^ ^33^, ^34^ ^]^ Complementary tests involving electrolyte temperature variations aligned with the applied laser power (Figure 3h; Figure S9b, Supporting Information) revealed only a marginal increase in AOR peak current density, further reinforcing the hot‐carrier‐driven mechanism. Comparative analysis of dark AOR currents under controlled thermal conditions and plasmonic excitation conclusively demonstrated that the kinetic‐controlled enhancements are attributable to LSPR‐induced hot carriers, rather than to thermal effects. Moreover, given that plasmonic excitation inherently involves two primary energy pathways namely photonic (non‐thermal) and thermal effects, the photonic contribution can be isolated by subtracting the thermal effect from the overall plasmonic response (Figure 3i). While localized heating and photothermal effects have been widely investigated and are known to contribute to photo(electro)catalytic activity,^[^ ^38^, ^39^ ^]^ a stronger correlation is observed between the net plasmonic effect and AOR activity against the individual thermal and photonic contributions, highlighting the synergistic nature of plasmon‐enhanced electrocatalysis.

### 2.3 Plasmon‐Modulated Interfacial Water During AOR Process

**Figure 4:**
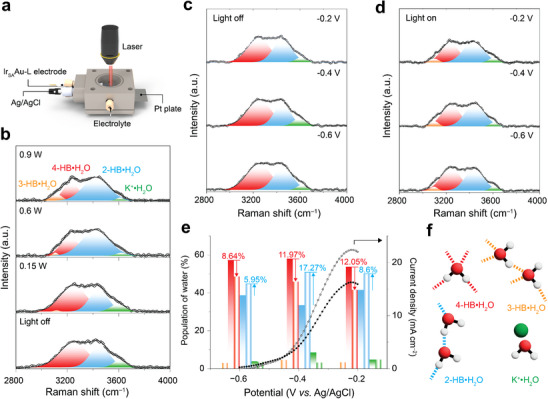
In situ Raman analysis of Ir_SA_Au‐L under plasmon‐assisted electrochemical conditions. In situ Raman spectra on interfacial water during AOR. a) Schematic drawing of electrochemical Raman cell. b) In situ Raman spectra of interfacial water at OCV without plasmonic irradiation and gradual increase of plasmonic irradiation power from 0.15 W to 0.9 W. Gaussian fit of three O–H stretching modes are shown in red, blue, and green, respectively. Emergence O–H stretching mode at lowest wavelength (orange) after plasmonic irradiation. Potential‐dependent Raman analysis without c) and with d) plasmonic irradiation (0.9 W). e) Analyses of potential‐dependent population of interfacial water from in situ Raman spectra and AOR current density (recorded in an electrochemical cell). f) Illustration of typical HB structures reported for liquid water at room temperature.

Understanding the interaction between reactants and water molecules on the Ir_SA_Au‐L catalyst, particularly under plasmonic illumination, is crucial for unraveling the AOR mechanism. To investigate this, we employed in situ Raman spectroscopy to examine how plasmonic irradiation influences the structural dynamics of interfacial water, a key factor in modulating reaction pathways. This approach allowed us to assess the strength and arrangement of the water HB network, which is crucial for orienting reaction intermediates, stabilizing transition states, and reducing energy barriers, thus enhancing the targeted reaction kinetics.^[^ ^40^ ^]^ Using a custom‐designed electrochemical Raman cell (**Figure** **4**a), we probed the structural ordering of interfacial water on Ir_SA_Au‐L at open‐circuit voltage (OCV) (Figure 4b; Figure S10, Supporting Information), both with and without plasmonic irradiation, focusing on O–H stretching modes (3000–3800 cm^−1^). Under OCV conditions, three distinct O–H stretching vibrations were identified, corresponding to different hydrogen‐bonded water structures namely 4‐coordinated hydrogen‐bonded water (4‐HB·H_2_O), 2‐coordinated hydrogen‐bonded water (2‐HB·H_2_O), and K^+^‐hydrated water (K^+^·H_2_O).^[^ ^41^, ^42^, ^43^, ^44^ ^]^ After plasmonic excitation, a new peak corresponding to a three‐coordinated hydrogen‐bonded water structure (3‐HB·H_2_O) emerged, indicating dynamic restructuring of the HB network.^[^ ^44^ ^]^ In addition, the observed reduction in K^+^·H_2_O population under increased plasmonic excitation suggests that localized surface plasmon resonance (LSPR) on Au induces a partial positive potential, creating charge repulsion between the positively charged Au surface and K^+^·H_2_O, thus decreasing K^+^ hydration levels (Figure S10d, Supporting Information). The presence of cations such as Na^+^ and K^+^ around active sites increases the flexibility of the hydrogen bonding (HB) network in aqueous solutions, which can hinder efficient dehydrogenation processes.^[^ ^45^ ^]^ From this perspective, our findings suggest that under plasmonic conditions, reduced hydration of K^+^ ions minimizes the proximity of hydrogen to the reactants. This effect promotes dehydrogenation and accelerates ammonia oxidation reaction (AOR) kinetics, highlighting the role of ionic hydration in modulating reaction dynamics through plasmonic excitation.

To deepen the understanding of the plasmonic effects with water orientation, potential‐dependent Raman analysis at various potential regions was conducted (Figure 4c,d). Plasmonic irradiation reduced the proportion of tetrahedral 4‐HB·H_2_O configuration of 8.64%, 11.97%, and 12.05% at −0.6, −0.4, and −0.2 V versus Ag/AgCl, respectively (Figure 4e). Under plasmonic irradiation, interestingly, the generation of a more flexible hydrogen network, characterized by 3‐HB·H_2_O, likely stabilizes reaction intermediates and reduces the activation energy for deprotonation by facilitating the reorientation of interfacial water.^[^ ^46^ ^]^ This shift is corroborated by a 27.26% increase in AOR peak intensity, emphasizing the role of interfacial water effects in enhancing the AOR process. Furthermore, a 17.27% increase in the population of 2‐HB·H_2_O (indicative of a weaker HB network) was also observed at −0.4 V, suggesting a shift toward a more flexible HB network under plasmonic conditions. This flexibility likely enhances the transport of NH_3_ and OH^*^ species at the electrode/electrolyte interface, leading to higher collision rates and faster AOR kinetics, as reflected in the increased current density observed at −0.4 V versus Ag/AgCl. The decreased presence of cation‐related hydrogen‐bonded water (K^+^·H_2_O) also indicates the beneficial influence of LSPR on AOR, as the extended K^+^·H_2_O bond length under positive potentials is favorable for AOR activity, forming a more dynamic structure.^[^ ^46^ ^]^ Moreover, Shen reported that more negative potentials orient water molecules with their H–ends toward the catalyst surface, leading to an increase in free–OH groups. This orientation results in H–down configurations and the formation of dangling bonds, which disrupt the deprotonation in HB network. In contrast, our observations toward positive potentials under plasmonic irradiation reveal a decrease in free–OH groups as the electric field directs water molecules with their H–ends away from the catalyst surface. Our findings highlight a consistent underlying mechanism by which the electric field governs the orientation of water molecules and modulates the HB network.^[^ ^41^ ^]^ Taken together, the decreased population of cation‐related weakly hydrogen‐bonded water and the small emergence of three‐hydrogen‐bonded network under plasmonic irradiation accelerate AOR kinetics and support intermediate stabilization.

Meanwhile, to further elucidate the dynamic evolution of the targeted material (Ir_SA_Au‐L) under operational conditions, in situ XAS measurements were performed using a custom three‐electrode electrochemical cell. The Ir *L_3_*‐edge XANES spectra (Figure S11a, Supporting Information) under varied potentials indicate that Ir_SA_Au‐L exhibited its highest white line intensity at the onset potential (−0.4 V versus Ag/AgCl) under plasmonic irradiation, with a gradual decrease at −0.25 V versus Ag/AgCl. This trend suggests that at −0.4 V versus Ag/AgCl, increased electron vacancies were formed at Ir sites, facilitating electron transfer from Ir to neighboring atoms and adsorbed oxygen species. This electron redistribution implies a favorable electronic restructuring of Ir_SA_Au‐L under plasmonic conditions, accelerating AOR kinetics.^[^ ^47^ ^]^ The FT of the Ir‐*L_3_* edge EXAFS spectra (Figure S11b, Supporting Information) further reveals a peak at ≈1.6 Å, representing the first‐shell Ir–O coordination. Under plasmonic irradiation, the Ir–O peak intensity progressively increased from OCV to −0.25 V, while the Ir─O bond distance decreased, indicating a local structural reconfiguration essential for enhanced reactivity. The increased intensity points to the self‐optimization and enhanced adsorption of reactive species under AOR conditions. Additionally, the decreasing Ir–O distance at OCV condition under plasmonic irradiation indicates that the LSPR leads to the reduction of Ir ionic radius, thereby accelerating deprotonation with ^*^OH during oxidative electrolysis (Figure S11c, Supporting Information).^[^ ^48^, ^49^ ^]^ Together, our in situ Raman and XAS data demonstrate that plasmonic irradiation induces a dual optimization of both the hydrogen‐bond network and Ir–O coordination on a chiral Au substrate. This synergy between interfacial water restructuring and shrinkage of Ir–O configurations drives a 28% increase in AOR activity compared to non‐plasmonic conditions, emphasizing LSPR pivotal role in modulating interfacial water structures and stabilizing Ir configuration for improved and stable AOR activities.

### 2.4 Theoretical Calculation

**Figure 5:**
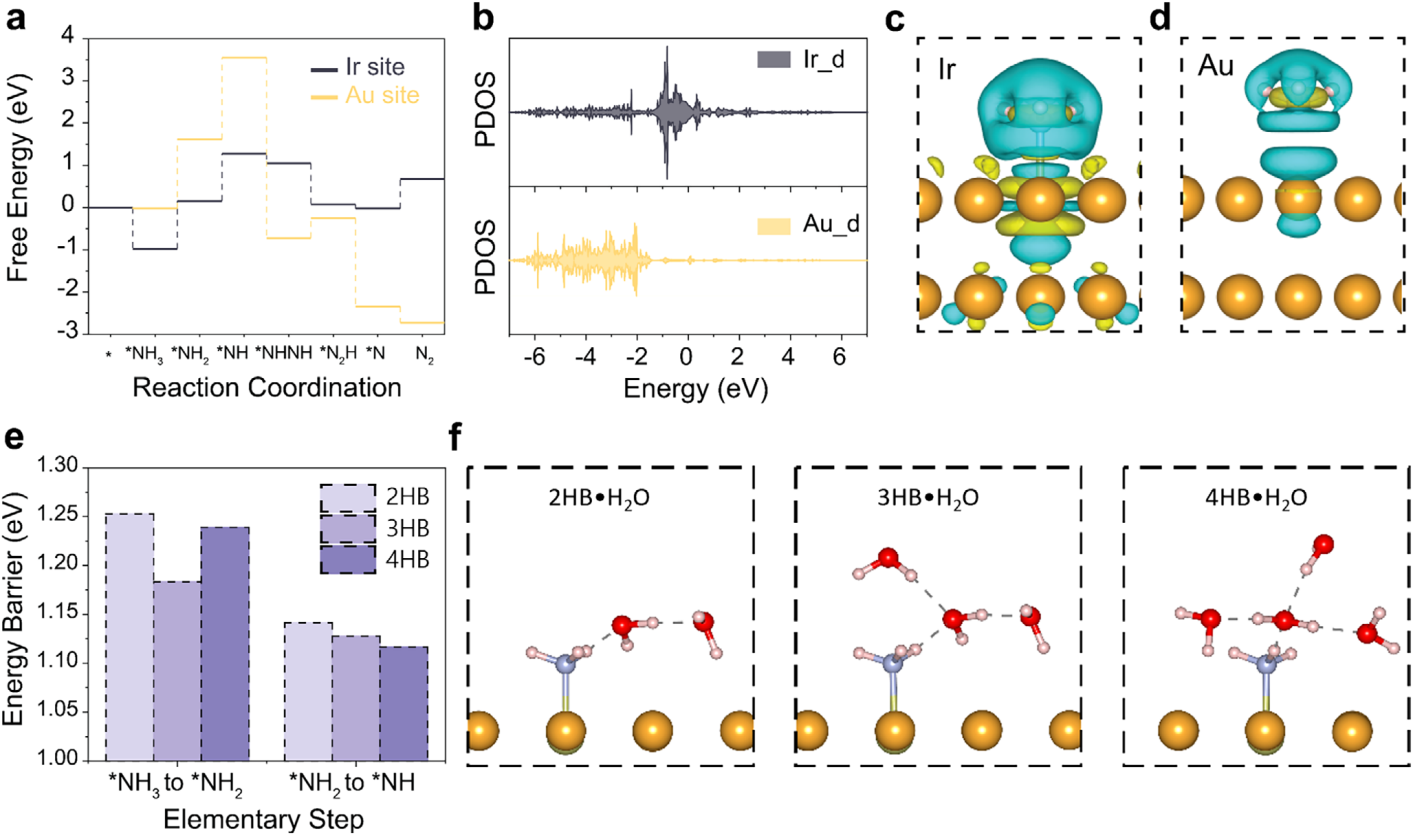
Theoretical calculations of reconfiguration of interfacial water structures during the AOR process under plasmonic illumination. a) Energy profiles of ammonia oxidation reaction on the Ir site and Au site in Ir_SA_@Au(111). b) Projected density of states (DOS) of *d* orbitals of the Ir site and Au site in Ir_SA_@Au(111). Charge difference between the adsorbed NH_3_ molecule and c) the Ir site and d) the Au site in Ir_SA_@Au(111), respectively, where the yellow part denotes the electron increase while the blue part denotes the electron decrease. e) Energy barriers of the first two hydrogenation steps within the frame of various hydrogen bond configurations. f) The corresponding optimized structures of different hydrogen bond configurations.

Density functional theory (DFT) calculations further elucidate the enhanced AOR catalytic performance of Ir_SA_Au‐L and the role of hydrogen bonding in the AOR process. Energy profiles identified the Ir single atom as the active site, exhibiting a lower energy barrier (1.13 eV) for the potential‐determining step (PDS) against the Au site (1.94 eV) on Au (111) (**Figure** **5**a). The high activity of the Ir site was attributed to an upshift in its *d*‐band center (−1.04 eV) relative to the Au site (−3.29 eV) (Figure 5b). According to *d*‐band center theory, this upshift enhances NH_3_ adsorption, facilitating effective electron transfer from NH_3_ to the Ir active site. As shown in Figure 5c,d, this strong electron transfer imparted a positive charge to the adsorbed NH_3_, promoting the subsequent dehydrogenation process. Energy profiles (Figure 5e) further identified the rate‐determining step in AOR as the first two dehydrogenation steps, both associated with high energy barriers. To investigate the influence of interfacial hydrogen bonds (HBs), the explicit water model was employed for these steps (Figure 5f). Computational results revealed that all possible interfacial HB structures effectively lowered the energy barrier for the second dehydrogenation step, making it more favorable than the first. Among the configurations, 3HB·H_2_O exhibited the lowest energy barrier (1.18 eV) compared to 2HB·H_2_O (1.25 eV) and 4HB·H_2_O (1.24 eV), aligning well with experimental findings.

### 2.5 LEDs‐Driven Plasmon‐Assisted Symmetric Wastewater Electrolyzer (LEDs‐PSWE)

**Figure 6:**
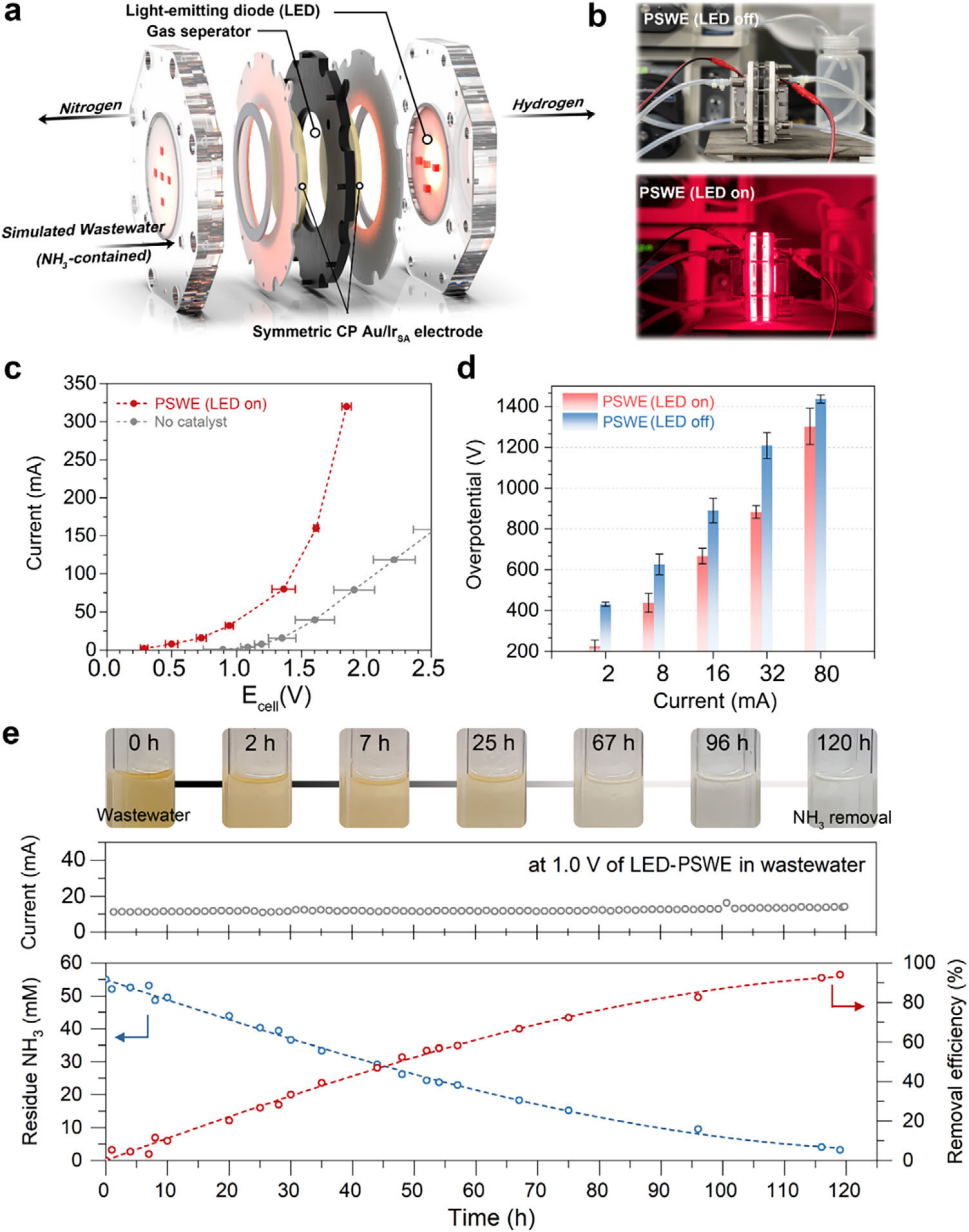
LEDs‐driven plasmon‐assisted symmetric wastewater electrolyzer (PSWE). a) Schematic diagram of continuous ammonia removal with producing H_2_ using an LEDs‐driven PSWE. b) Photograph of the LEDs‐driven PSWE under light‐off (upper) and light‐on (bottom). c) I–V curves of LEDs‐driven PSWE for Ir_SA_Au‐L electrode and without catalyst. d) The overpotential for Ir_SA_Au‐L‐based LEDs‐PSWE system under the light on/off corresponding to the different current windows. e) The overall current and NH_3_ removal efficiency under continuous electrolysis at 1.0 V. The upper shows a photograph of Nessler's reagent‐treated wastewater corresponding to different elapsed times on the stability test.

We further extend our chiral plasmonic electrocatalysts by integrating antenna‐reactor systems powered by light‐emitting diodes (LEDs) to drive visible‐light‐assisted wastewater electrolysis. This approach, demonstrated using CP Ir_SA_Au‐L, enables simultaneous water treatment and hydrogen production within an LED‐based plasmon‐assisted symmetric wastewater electrolyzer (LEDs‐PSWE) (**Figure** **6**). The system incorporated a customized LED chip emitting at a 650 nm wavelength, aligned parallel to the electrodes (Figure 6a), enabling efficient light utilization and enhanced reaction performance. The NH_3_‐contained simulated wastewater (55 mm NH_3_) continuously fed into LEDs‐PSWE system, and the performances were investigated under LED light on/off (Figure 6b). The current–voltage (I–V) profile demonstrated remarkably improved current (×40 times) against without plasmonic electrocatalyst at 0.9 V (Figure 6c). When LED illumination was absent, the onset potential increased by 200 mV, with the overpotential rising by an average of 215 mV across current ranges from 2 to 80 mA (Figure 6d; Figure S12, Supporting Information). Additionally, we investigated residual ammonia concentration and removal efficiency as a function of operation time during continuous wastewater treatment using the LEDs‐PSWE system with the CP Ir_SA_Au antenna‐reactor catalyst (Figure 6e; Figure S13, Supporting Information). The ammonia concentration steadily decreased over time, achieving an average removal efficiency of 94.0% and stable electrolysis current over 120 h. The color of the treated wastewater transitioned from yellow to colorless during the stability test, visually confirming the treatment progress (Figure S14, Supporting Information). This study presents a proof‐of‐concept using Ir single atom on chiral Au substrates to elucidate the mechanistic role of plasmonic excitation in enhancing AOR activity, while the use of energy‐efficient LED illumination and the modular system architecture provides a viable foundation for scalability using earth‐abundant plasmonic materials (e.g., Cu, Al, or doped semiconductors). Moreover, the plasmon‐induced reorientation of interfacial water observed here may serve as a generalizable strategy to modulate charge transfer and reaction pathways in other electrocatalytic systems such as urea oxidation, nitrate reduction, OER, NRR, and CO_2_RR, where interfacial water structure plays a pivotal role in governing kinetics and selectivity.

## 3 Conclusion

This study demonstrates a mechanistic framework linking the modulation of hydrogen‐bond (HB) networks in interfacial water to the enhancement of AOR activity on the Ir_SA_Au‐L electrocatalyst under LSPR excitation. Our findings reveal that plasmonic effect induces a dynamic restructuring of interfacial water, converting rigid tetrahedral 4‐HB configurations into more flexible 3‐ and 2‐HB structures. This transformation, corroborated by in‐situ Raman spectroscopy and theoretical calculations, facilitates reactant stabilization, reduces deprotonation energy barriers, and accelerates AOR kinetics, leading to a 28% improvement in catalytic performance. Furthermore, our study clarifies the role of chirality in the catalytic process. While chiral Au substrates were employed for single‐atom Ir stabilization and active site optimization, we found that the primary contributor to AOR enhancement is the LSPR‐driven modulation of interfacial water rather than chirality‐induced spin selectivity. Operando XAS confirms that LSPR activation induces polarization at the single‐atom Ir sites, leading to Ir─O bond compression, which enhances catalytic efficiency and stability. By integrating this plasmonic mechanism into a practical LEDs‐driven AOR symmetric electrolyzer, we achieved 120 h of stable operation with 94% NH_3_ removal efficiency under landfill leachate‐like wastewater conditions. These insights highlight the potential of LSPR‐mediated interfacial water engineering for advancing electrochemical oxidation processes and developing energy‐efficient wastewater treatment technologies.

## Conflict of Interest

The authors declare no conflict of interest.

## Supporting information



Supporting Information

## Data Availability

The data that support the findings of this study are available from the corresponding author upon reasonable request.
