# Supplementary material for: Plasmon‐Driven Reorientation of Interfacial Water for Wastewater Electrolysis with Light‐Emitting Diode Illumination
Source: Adv Sci (Weinh). 2025 Jun 25;12(35):e07147. doi: 10.1002/advs.202507147 (PMC12463107; doi:10.1002/advs.202507147)
Supplement: Supplementary file 1 — Supporting Information [file ADVS-12-e07147-s001.docx]

Supporting Information

Plasmon-driven Reorientation of Interfacial Water for Wastewater Electrolysis with Light-emitting Diode Illumination

Nur Aqlili Riana Che Mohamad^†[a]^, Kyunghee Chae^†[a]^, Qiang Zhou^[b],[c]^, Wen-Tse Huang^[d]^, HyunJeong Lee^[a]^, Jaehyun Son^[e]^, Jooho Moon^[e]^, Yunfei Bu^[f]^, Feng Gong*^[c]^, Ru-Shi Liu*^[d]^, Jeongwon Kim*^[a],[g],[h]^, and Dong Ha Kim*^[a],[g],[i],[j],[k]^

[a] Dr. N.A.R. Che Mohamad, K. Chae, H. Lee, Dr. J. Kim and Prof. D. H. Kim
Department of Chemistry and Nanoscience,
Ewha Womans University,
Seoul 03760, Republic of Korea
E-mail: [dhkim@ewha.ac.kr](mailto:dhkim@ewha.ac.kr)

[b] Q. Zhou
School of Engineering,
The University of Tokyo,
7-3-1 Hongo, Bunkyo-ku, Tokyo 113-8656, Japan

[c] Q. Zhou and Prof. F. Gong
Key Laboratory of Energy Thermal Conversion and Control of Ministry of Education,
School of Energy and Environment,
Southeast University, Nanjing, 211189, Jiangsu, China
E-mail: [gongfeng@seu.edu.cn](mailto:gongfeng@seu.edu.cn)

[d] Prof. W. -T. Huang and Prof. R. -S. Liu
Department of Chemistry,
National Taiwan University,
106 Taipei, Taiwan
E-mail: [rsliu@ntu.edu.tw](mailto:rsliu@ntu.edu.tw)

[e] J. Son and Prof. J. Moon
Department of Materials Science and Engineering,
Yonsei University,
50 Yonsei-ro Seodaemun-gu, Seoul 03722, Republic of Korea

[f] Prof. Y. Bu
UNIST-NUIST Research Center of Environment and Energy (UNNU), School of Environment Science and Technology
Nanjing University of Information Science and Technology,
Nanjing 210044, People’s Republic of China

[g] Dr. J. Kim and Prof. D. H. Kim
Nanobio·Energy Materials Center (National Research Facilities and Equipment Center),
Ewha Womans University,
Seoul 03760, Republic of Korea

[h] Dr. J. Kim
Department of Chemistry,
Northwestern University,
Evanston, IL, USA
E-mail: [jeongwon.kim@northwestern.edu](mailto:jeongwon.kim@northwestern.edu)

[i] Prof. D. H. Kim
College of Medicine,
Ewha Womans University,
25, Magokdong-ro 2-gil, Gangseo-gu, Seoul 07804, Republic of Korea

[j] Prof. D. H. Kim
Graduate Program in Innovative Biomaterials Convergence,
52 Ewhayeodae-gil, Seodaemun-gu, Ewha Womans University, Seoul 03760, Republic of Korea

[k] Prof. D. H. Kim
Basic Sciences Research Institute (Priority Research Institute),
Ewha Womans University,
Seoul 03760, Republic of Korea)

**Experimental section**

**Fabrication of Ir_SA_Au-L electrocatalyst**

Achiral and chiral gold electrodepositions were performed using a three-electrode configuration, where titanium felt served as the working electrode, a graphite rod as the counter electrode, and an Ag/AgCl electrode as the reference. The electrodeposition solution comprised 2 mM HAuCl_4_·3H_2_O, 0.1 M KCl, and 0.05 mM DL/D/L-cysteine, maintained at room temperature. Deposition was conducted via constant-potential electrolysis at 0 V vs. Ag/AgCl for 300 seconds using an Autolab potentiostat. The resulting Au-L NF/Ti electrode was thoroughly rinsed with deionized water and air-dried at ambient conditions. Single atom Ir deposition on the Au-L NF/Ti was carried out under identical three-electrode conditions, using Ag/AgCl as the reference electrode. The deposition solution contained 100 μM IrCl_4_ and 1 M KOH at room temperature. Electrochemical deposition was achieved by cycling the potential between 0.10 V and -0.40 V vs. RHE for 20 cycles at a scan rate of 5 mV s^–1^. The electrode was subsequently rinsed with deionized water and dried at ambient temperature.

**Computational calculations**

All spin-polarized computations were performed under the framework of density functional theory (DFT). Perdew-Burke-Ernzerhof (PBE) functional among a generalized gradient approximation (GGA) was introduced to describe the exchange and correlation effect of electron. The projector augmented wave (PAW) method was employed to describe the electron-ion interaction. The kinetic energy cutoff for plane wave was set to 400 eV. The Brillouin zone was sampled by a 3× 3× 1 grid for structure relaxation and 5× 5× 1 for the calculation of density of states (DOS). The criterion of structure relaxation was set to 10^-5^ eV for total energy and 0.05 eV/Å for the force of each atom. DFT-D3 method was employed for the correction of vdWs force. The Ir_SA_@Au(111) catalyst was modelled by a three-layer 2× 2× 1 supercell of Au(111) surface with a replaced Ir atom to simulate Ir single atom.

**Materials characterization**

Morphological analysis was performed using high-resolution scanning electron microscopy (HR-SEM, JSM-7610F) and transmission electron microscopy (TEM, JEM-2100F). High-angle annular dark-field scanning TEM (HAADF-STEM) and energy-dispersive X-ray spectroscopy (EDS) were conducted on a FEI TitanTM 80-300 microscope to investigate structural and compositional details. Powder X-ray diffraction (PXRD) patterns were recorded on a Rigaku Dmax 2000 diffractometer with Ni-filtered Cu-Kα radiation (λ = 1.5418 Å) over a 2θ range of 20°–80° under air-free conditions. Surface chemical states were analyzed using X-ray photoelectron spectroscopy (XPS) on a Thermo Scientific K-Alpha system equipped with a dual-beam source. X-ray absorption spectroscopy (XAS) was performed at beamlines TPS 44A and TLS 17C1 at the National Synchrotron Radiation Research Center (NSRRC), Taiwan. Circular dichroism (CD) spectra were recorded using a JASCO J-1500 spectrometer. Magnetic conductive atomic force microscopy (mc-AFM) was conducted on a SPA400 system (Seiko Instruments) over a 2 μm × 2 μm area using a Co-Cr-coated cantilever pre-magnetized for 60 minutes. I-V characteristics were measured by sweeping the voltage from -1.5 V to +1.5 V at 0.5 Hz, with 30 sweeps averaged across multiple sample locations.

**Electrochemical performance measurement**

Electrochemical tests were conducted using Biologic SP-150e potentiostat (Biologic Science Instruments), with an Ag/AgCl electrode as the reference and a graphite electrode as the counter. A 0.05 mM NH_4_Cl in 0.5 M KOH was used as the electrolyte, and all potentials were calibrated to the RHE. The AOR activities was evaluated from CV curves recorded at a scan rate of 10 mV s^–1^. Ohmic resistance was determined through electrochemical impedance spectroscopy (EIS) over a frequency range of 1000 to 0.1 Hz to compensate for iR losses.

***In-situ* Raman and XAS measurement**

Electrochemical Raman measurements were performed using a confocal Raman microscope system, HORIBA. A 637.8 nm He-Ne laser and a 50× microscope objective with a numerical aperture of 0.55 were utilized for all measurements. Raman frequencies were calibrated against a Si wafer before each experiment. *In-situ* electrochemical Raman experiments were conducted in a custom-built Raman cell, with potential control achieved using a Biologic SP-150e potentiostat. Cobalt K-edge X-ray Absorption Fine Structure (XAFS) analyses were carried out at the Pohang Light Source (PLS-II) in Korea, where the storage ring operated at 10 GeV with a steady current of 3 kA. The XAFS data were recorded in fluorescence mode using ion chambers, followed by background subtraction and normalization with Athena software. *Operando* XAFS measurements were performed using a custom-designed cell. To capture the dynamics of reversible oxygen electrochemical reactions (charge/discharge, OCV mode) and plasmonic excitation (laser on/off mode), the system was equilibrated for 5 minutes prior to each measurement step as a conditioning procedure. XAFS data acquisition was completed within a single beam time session.

**LED-driven symmetric wastewater electrolyzer**

The LEDs-driven plasmon-assisted symmetric wastewater electrolyzer (LEDs-PSWE) system were performed in ambient pressure and without supplying external heating. A custom-configured red LED (CHANZON 100W) was integrated into the system using a quartz window-assembled electrolyzer. Electrodes with surface areas of 45 pi and 50 pi were fabricated through electrodeposition, adhering to the previously established protocol. The electrolyte mimicked landfill leachate, comprising 0.5 M KOH and 55 mM NH4+, with a flow rate controlled at 20 mL/min at the inlet of each electrolyzer. The outlet allowed for the discharge of both the electrolyte and the generated gases. For each experimental run, 200 mL of wastewater was loaded into the wastewater reservoir.


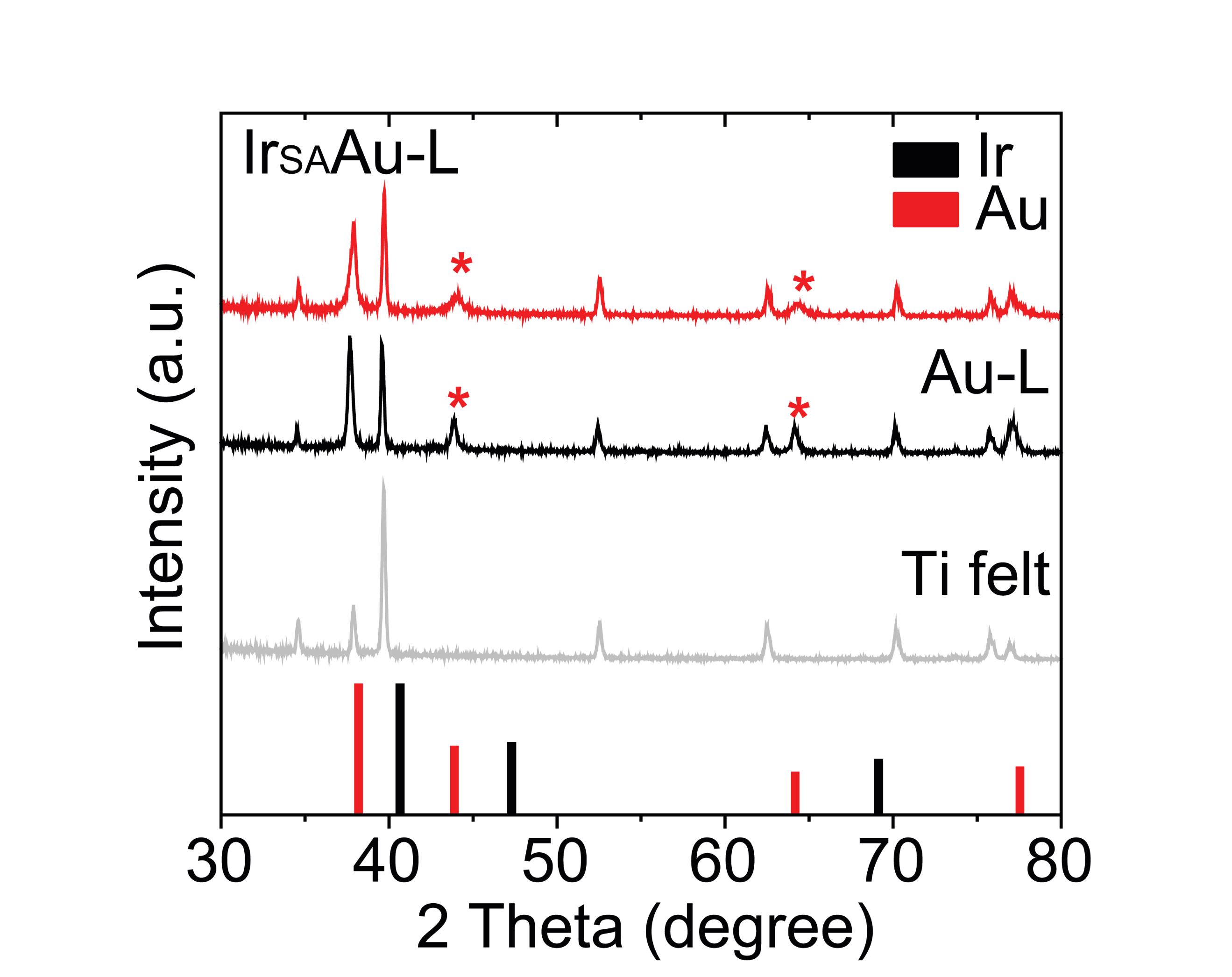


**Figure S1.** XRD patterns of Ti felt, chiral Au (Au-L) and Ir_SA_Au-L.


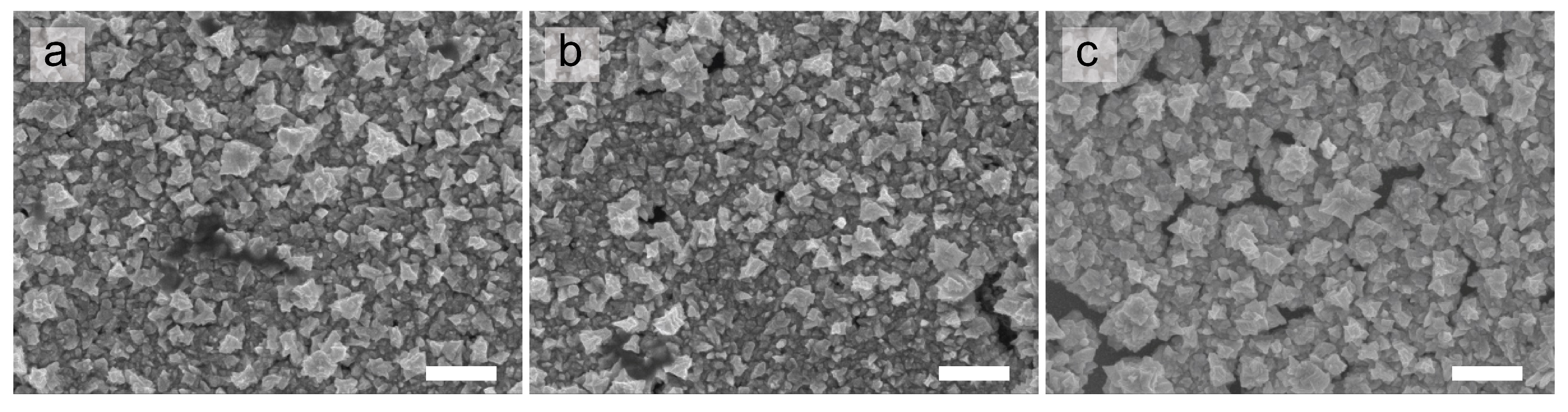


**Figure S2.** Scanning electron microscopy (SEM) image of (a) DL-Cysteine Au on Ti felt, (b) D-Cysteine Au on Ti felt and (c) L-Cysteine Au on Ti felt. The scale bar is 500 nm.


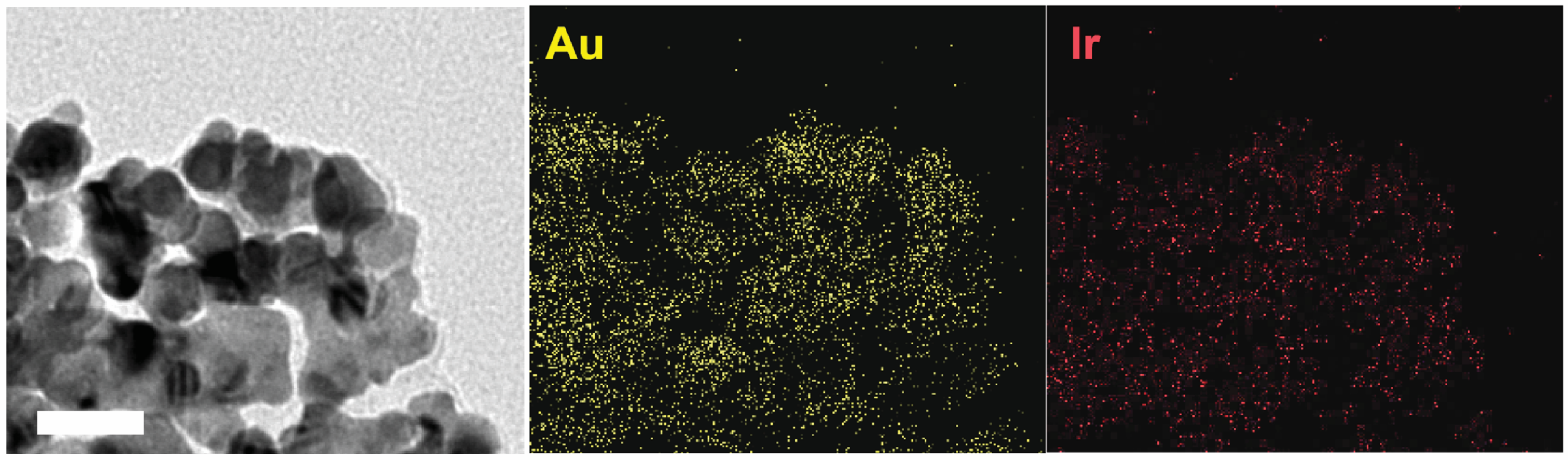


**Figure S3.** TEM images of Ir_SA_Au-L and corresponding EDS elemental mapping images. The scale bar is 10 nm.

**Figure S4.** (a) HAADF-STEM image of Ir_SA_Au-L and (b) intensity profiles along the dashed rectangles.


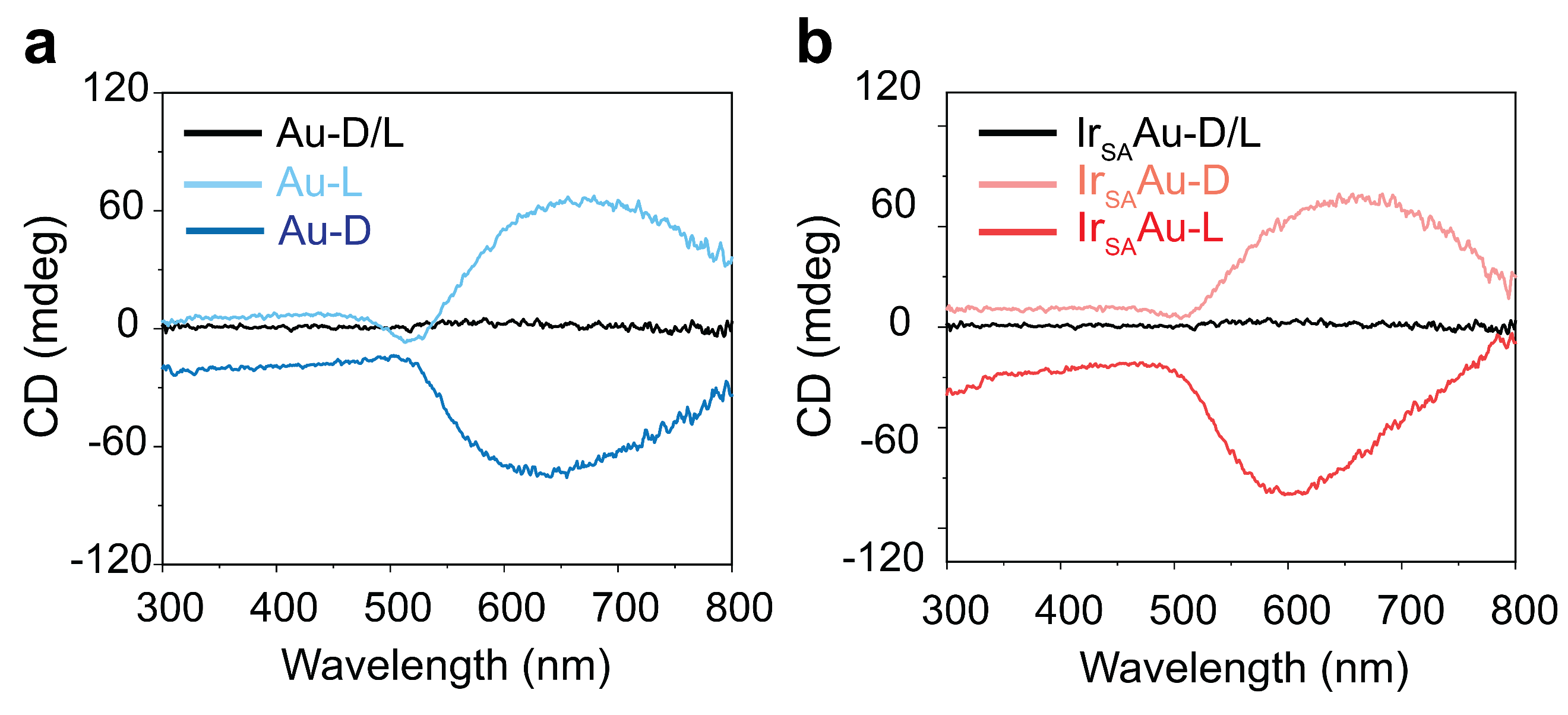


**Figure S5.** CD spectra of (a) Au-DL/D/L-cysteine and (b) Ir_SA_Au-DL/D/L-cysteine measure on an ITO substrate.
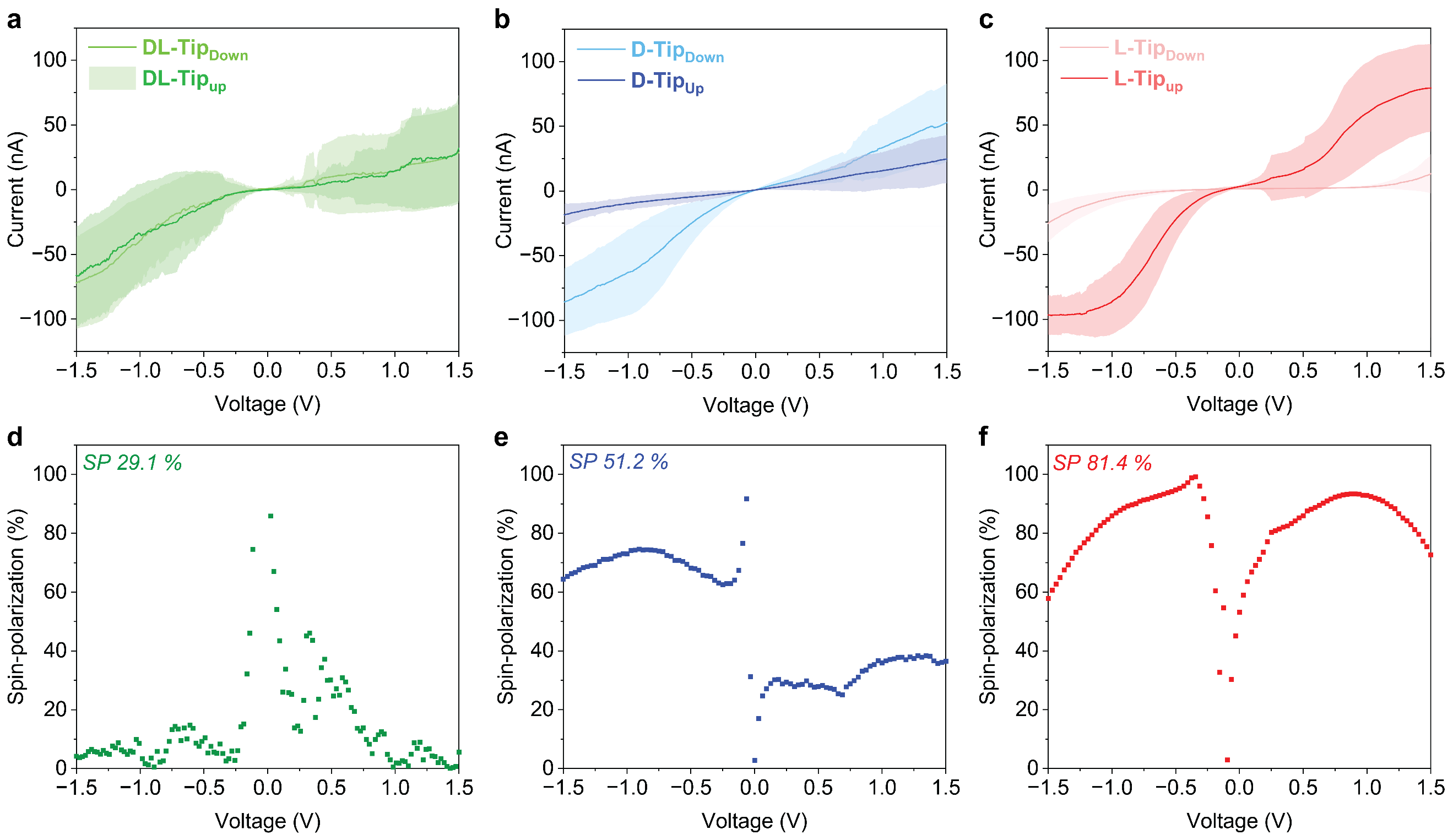


**Figure S6.** I-V curves of (a) Ir_SA_Au-DL cysteine, (b) Ir_SA_Au-D cysteine and (c) Ir_SA_Au-L cysteine films with spin polarization percentage (SP%) in the range of -1.5 to +1.5 V. The CoCr tip was magnetized along the upward or downward orientation. The average I-V curve recorded over 30 scans at different points is shown. Spin polarization percentage (SP%) of (d) Ir_SA_Au-DL, (e) Ir_SA_Au-D and (f) Ir_SA_Au-L films.


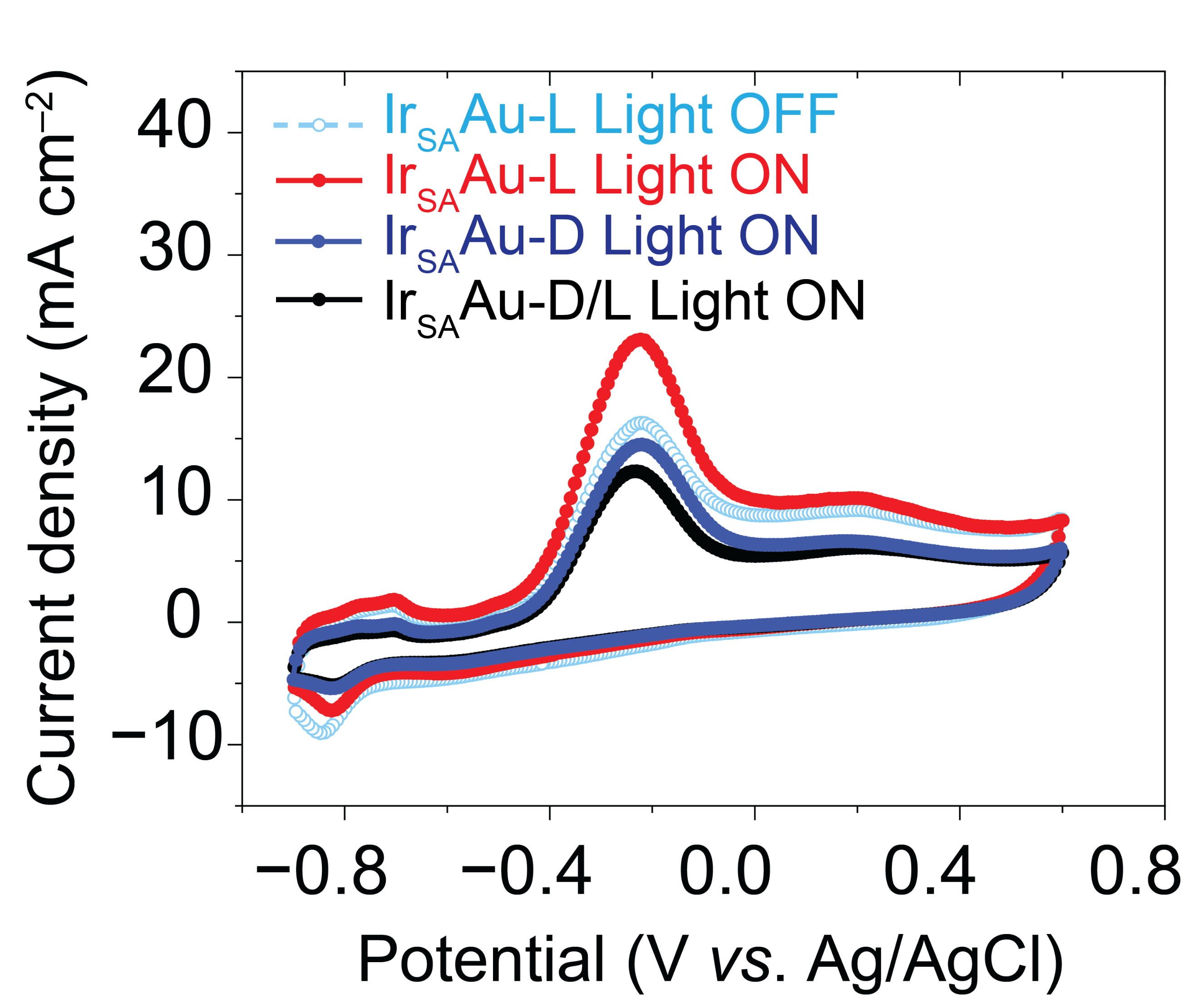


**Figure S7.** AOR CV curves of Ir_SA_Au with different cysteine configurations in 0.5 M KOH containing 55 mM NH_4_Cl electrolyte with and without illuminations.


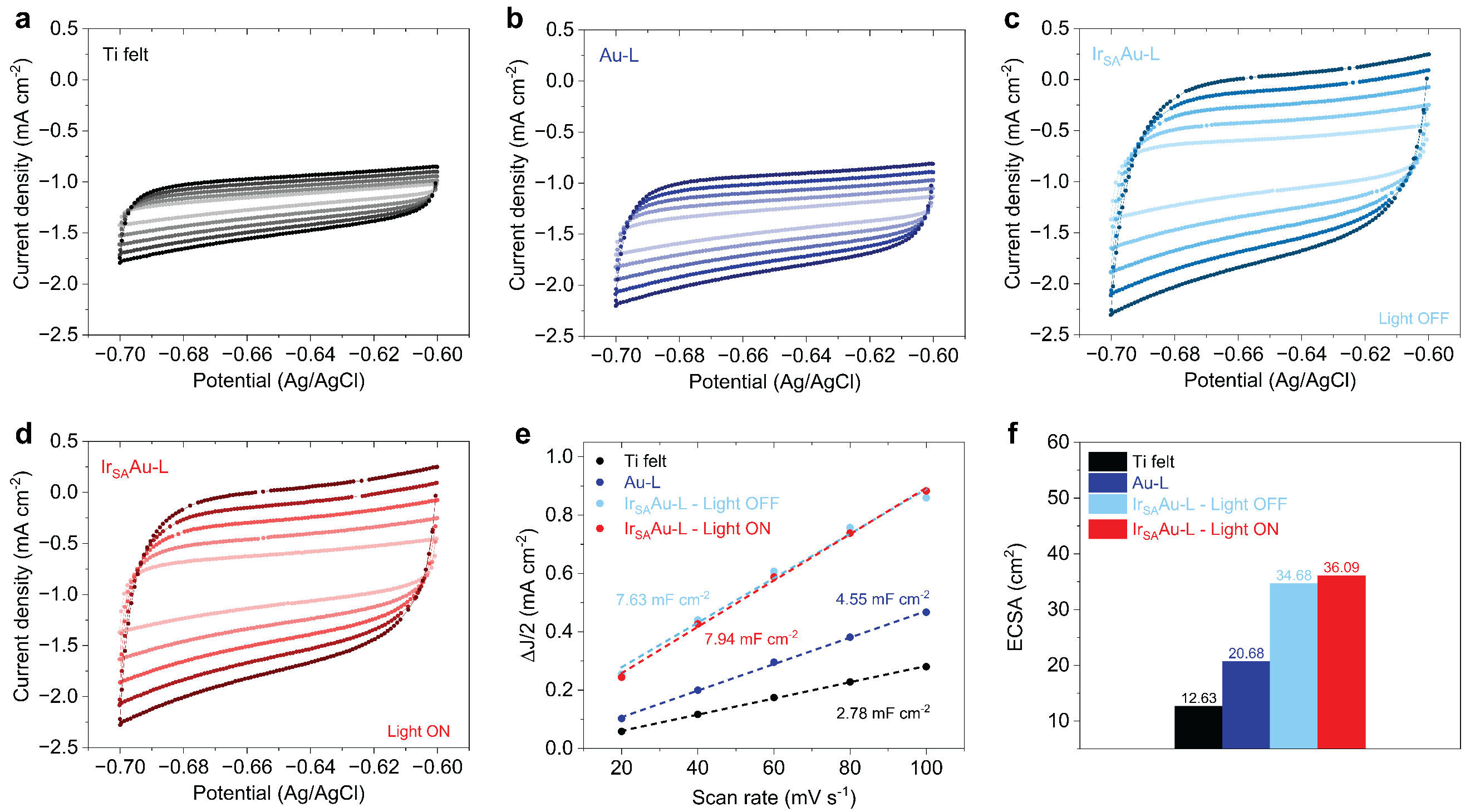


**Figure S8.** Electrochemical CV scans recorded for each electrode with scan rate of 20, 40, 60, 80, and 100 mV s^-1^ for (a) Au-L on Ti felt, (b) Au-L on Ti felt, (c) Ir_SA_Au-L on Ti felt under dark condition, and (d) Ir_SA_Au-L on Ti felt under plasmonic irradiation in 0.1 M KOH electrolyte containing 55 mM NH_4_Cl electrolyte. (e) Capacitive current of each electrode at - 0.65 V with different scan rates and (f) the corresponding calculated ECSA.


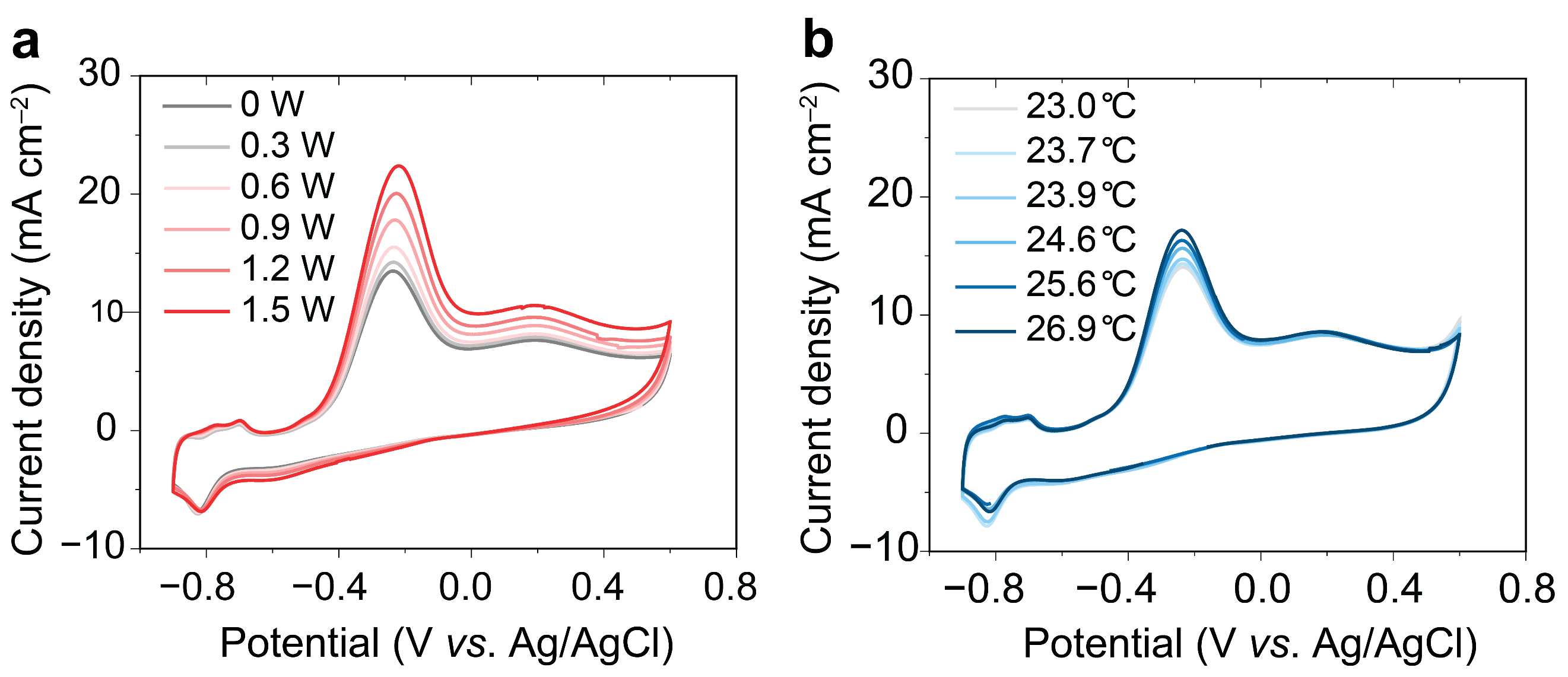


**Figure S9.** (a) AOR CV curves under different power laser irradiation and (b) different electrolyte temperatures (in accordance with the applied laser power).


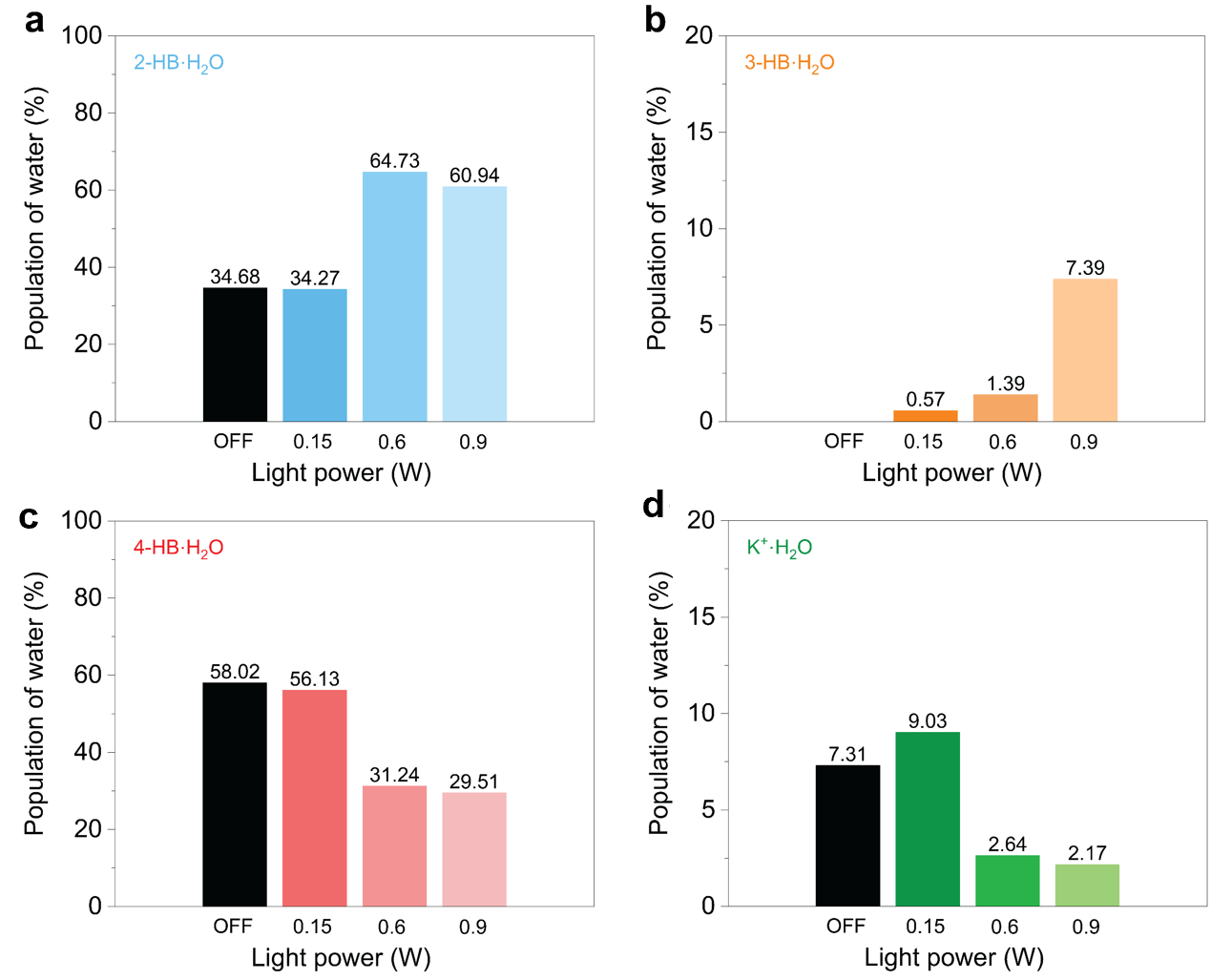


**Figure S10.** Laser power dependent population of interfacial water from *in-situ* Raman spectra**.** (a) 2-coordinated hydrogen bonded water. (b) 3-coordinated hydrogen bonded water. (c) 4-coordinated hydrogen bonded water. (d) K^+^-coordinated hydrated water.


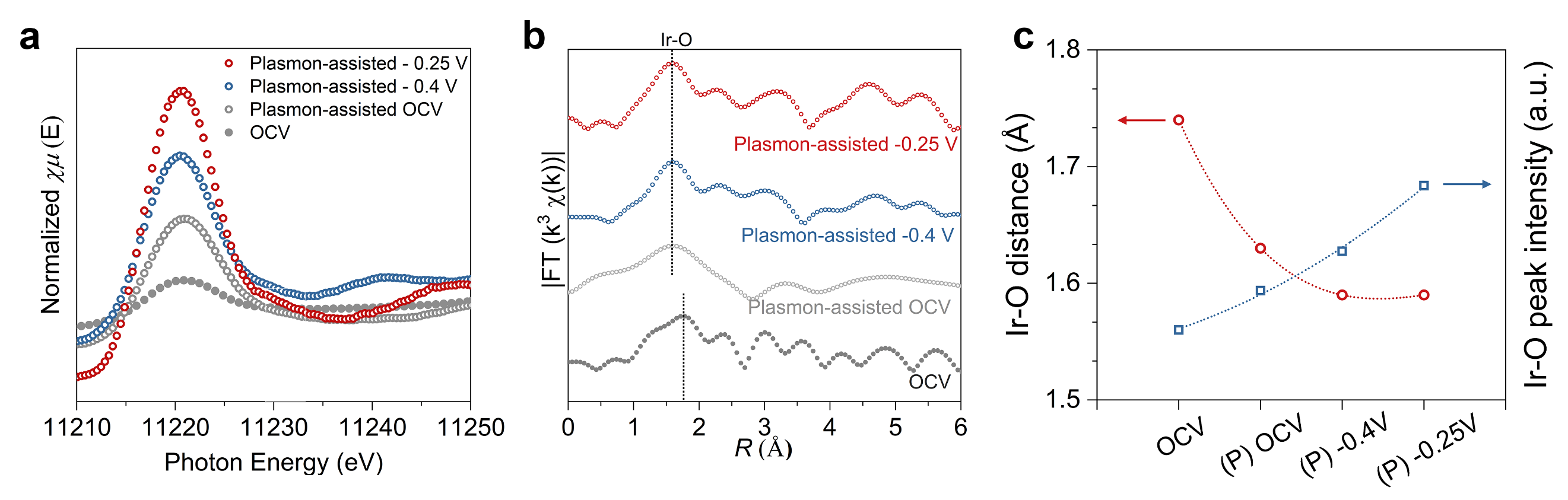


**Figure S11.** (a) *In-situ* XAS measurement during AOR of Ir_SA_Au-L under plasmonic illumination. (a) XANES spectra. (b) Fourier transform EXAFS spectra. Dash line represents Ir-O. (c) EXAFS determined Ir–O distance and Ir–O intensity as a function of applied potential and plasmonic irradiation.


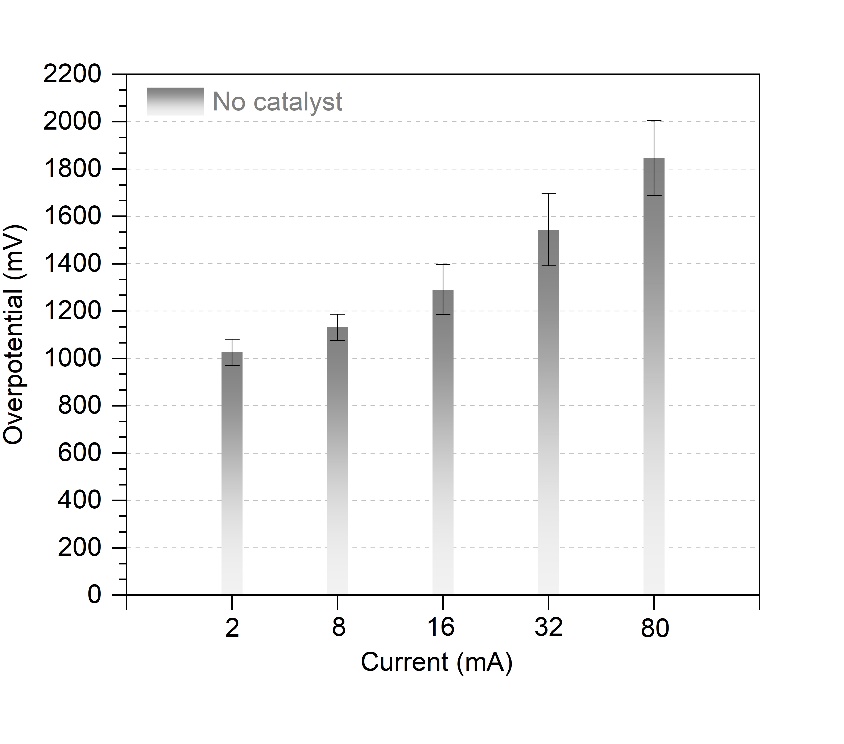


**Figure S12.** The overpotential without catalyst-based LEDs-PSWE system under light on/off corresponding to different current windows.


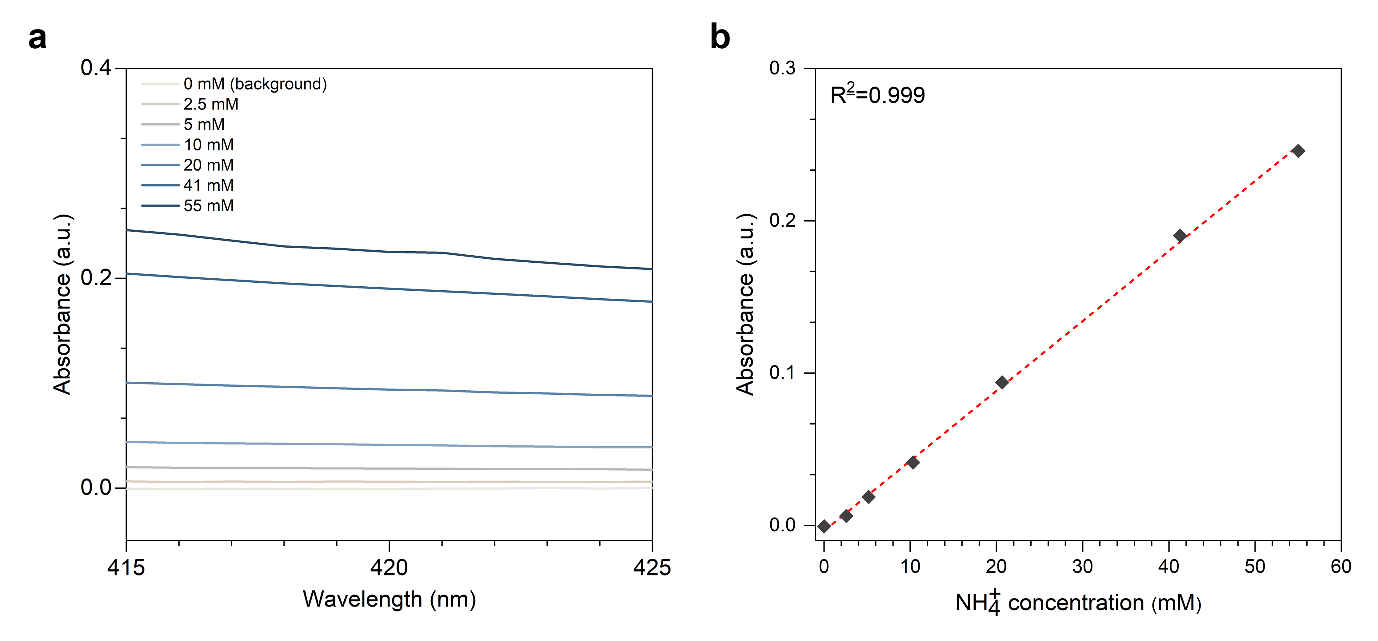


**Figure S13.** UV-vis measurements of the concentration of ammonium with Nessler’s reagents. (a) UV-vis spectra for standard solutions. (b) Calibration curve for ammonium concentration *vs*. absorbance. R^2^ = 0.9989


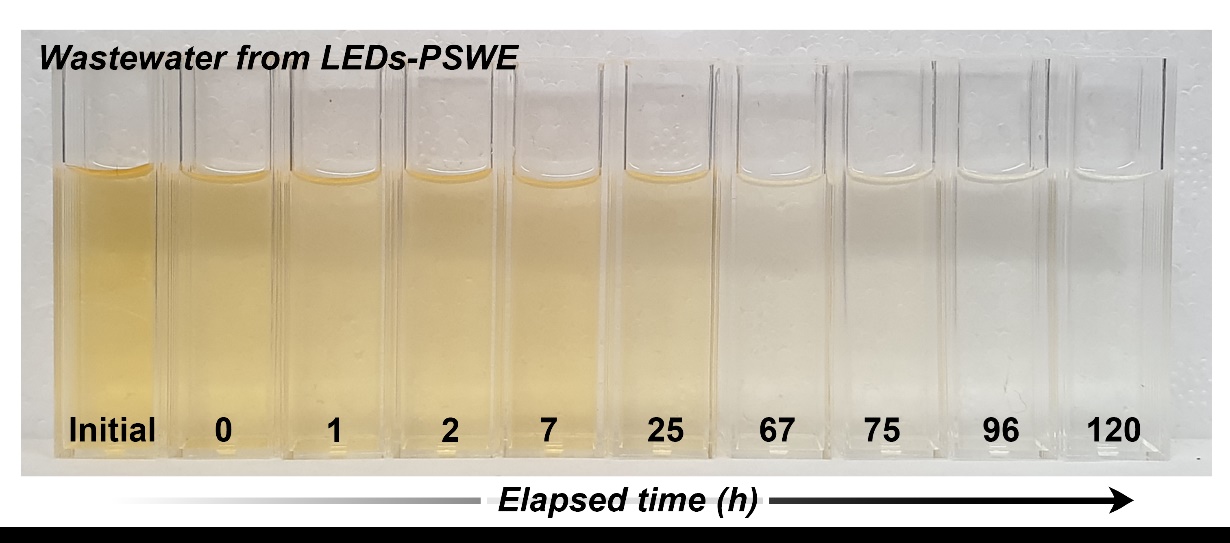


**Figure S14.** The photograph of Nessler reagent treated wastewater corresponding to different elapsed time on stability test. Wastewater electrolyte from LEDs-PSWE system during stability test at 1 V.
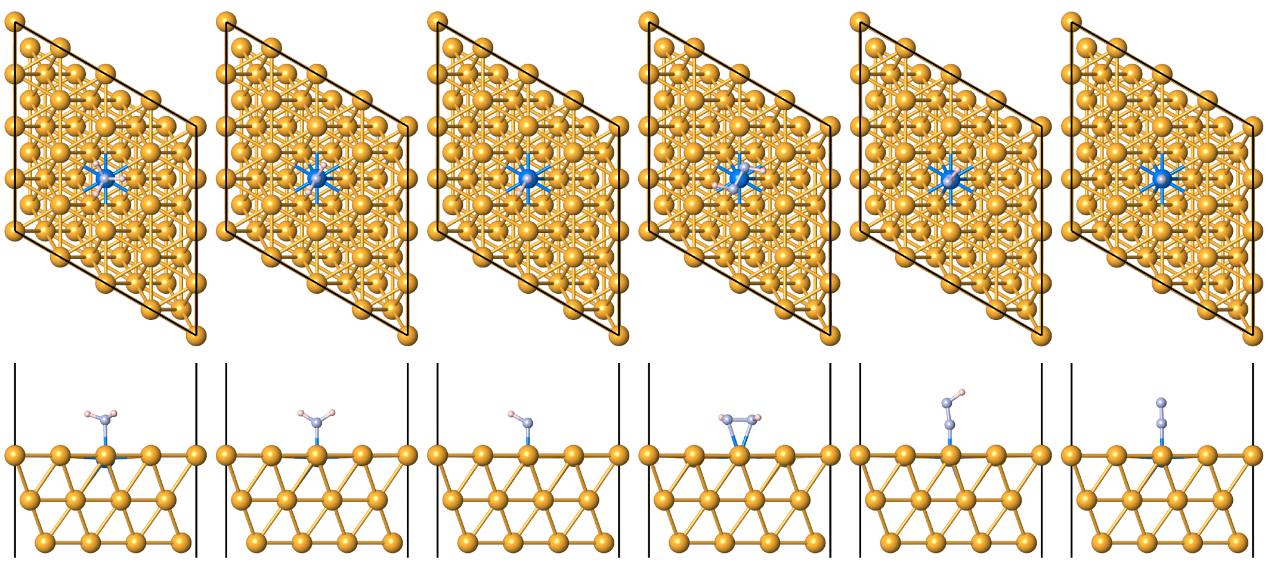


**Figure S15.** AOR on the Ir site.


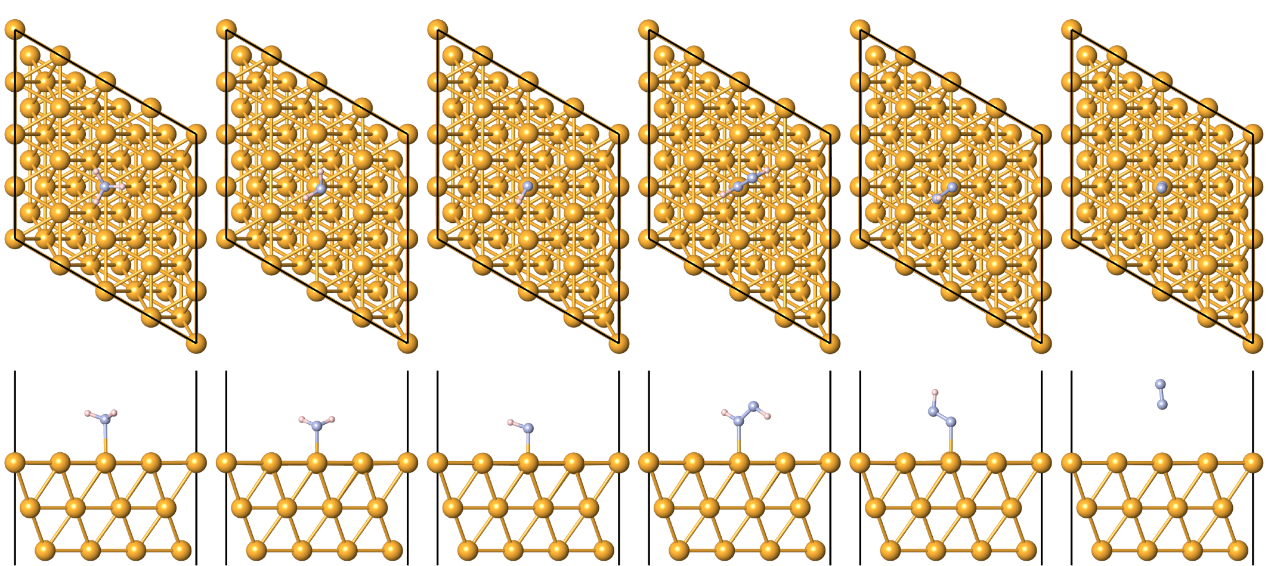


**Figure S16.** AOR on the Au site.


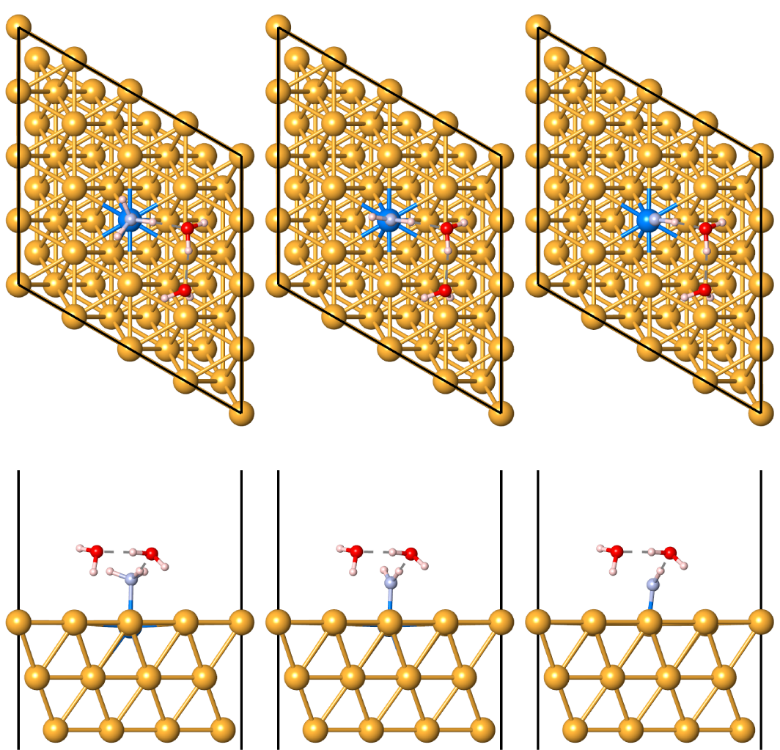


**Figure S17.** HB2 structures.


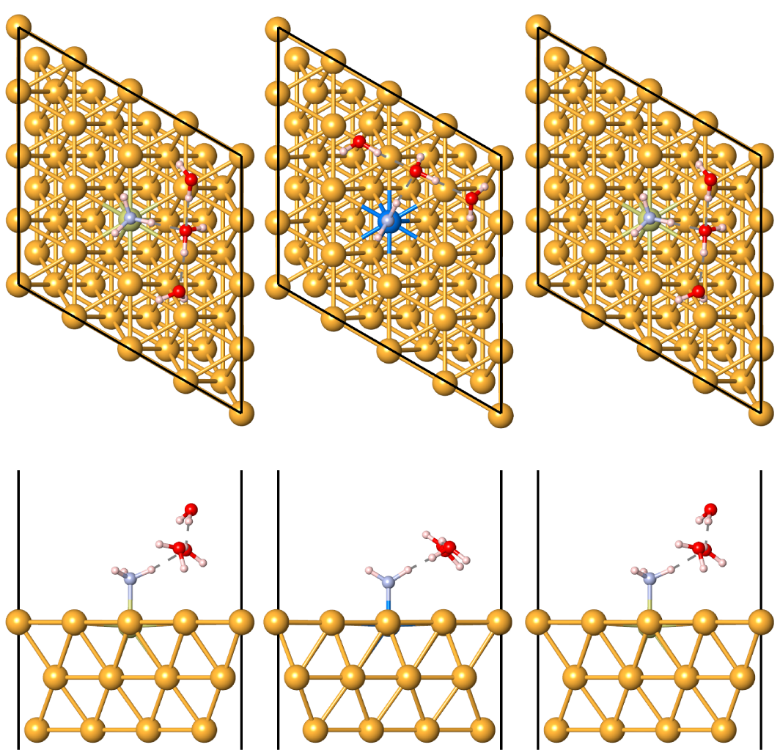


**Figure S18.** HB3 structures.


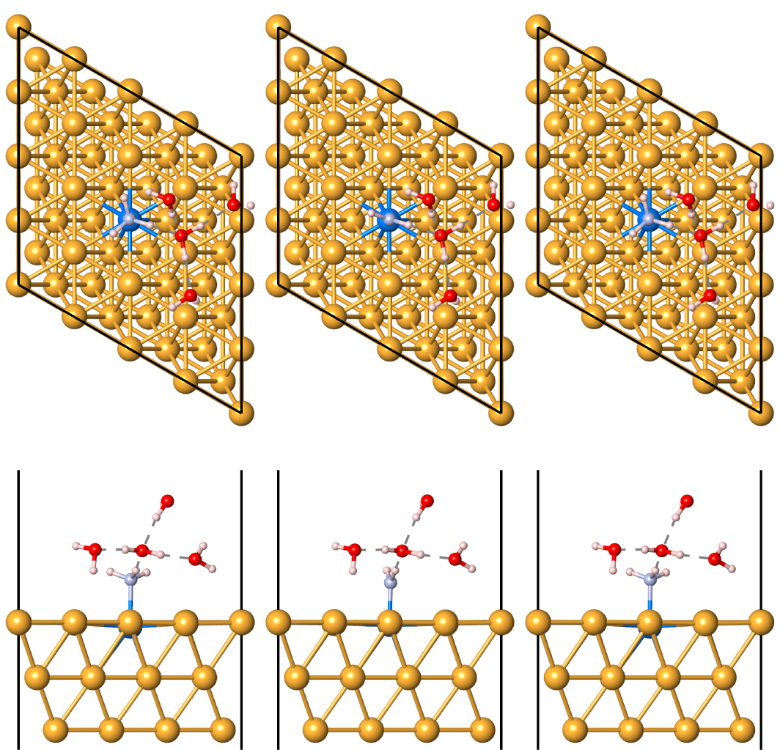


**Figure S19.** HB4 structures.

**Table S1.** Comparative performance of recent AOR electrocatalysts under comparable conditions.

| No. | Catalyst | Fabrication method | Current density (mA cm^–2^) | Electrolyte | Reference |
| --- | --- | --- | --- | --- | --- |
| 1 | Ir_SA_Au-L | Electrodeposition | 22.6 at -0.3 V_Ag_/_AgCl_ | 0.05 M NH_4_Cl + 0.5 M KOH | This work |
| 2 | Pt/C | Tanaka Kikinzoku Kogyo | 0.920 at -0.3 V_Ag_/_AgCl_ | 0.1 M NH_3_ + 1.0 M KOH | *Joule*, **2019**, *10*, 2472-2484 |
| 3 | PtIr/C | Premetek Co | ~8.5 at 0.6 V_RHE_ | 0.1 M NH_3_ + 1.0 M KOH | *Joule*, **2019**, *10*, 2472-2484 |
| 4 | Pt-NCs | Hydrothermal | ~5.0 at 0.66 V_RHE_ | 0.1 M NH_4_ + 1.0 M KOH | *J. Energy Chem.* **2020**, *47*, 234–240 |
| 5 | PtSnO_2_/C 100 | Alcohol-reduction process | ~0.7 at -0.2 V_Hg_/_HgO_ | 0.5 M NH_4_OH + 1.0 M KOH | *Appl. Catal. B: Environ.* **2020**, *264*, 118458 |
| 6 | Au@Pt NPs | Seed-mediated growth | ~1.3 at 0.7 V_RHE_ | 0.05 M NH_3_.H_2_O + 1.0 M NaOH | *Angew. Chem. Int. Ed*. **2020**, *59*, 18430 –18434 |
| 7 | Flower-like Pt | Electrodeposition | ~550 at -0.3 V_Ag_/_AgCl_ | 1.0 M NH_3_ + 5.0 M KOH | *J. Mater. Chem. A*, **2021**, *9*, 11571-11579 |
| 8 | La_0.5_Sr_1.5_Ni_0.9_Cu_0.1_O_4-δ_-Ar | Modified Pechini method | ~7.5 at -0.3 V_Ag_/_AgCl_ | 0.055 M NH_4_Cl + 0.5 M KOH | *Adv. Funct. Mater.* **2022**, *32*, 2204881 |
| 9 | Pt_3_Ru_1/2_Co_1/2_ | Hummers method | ~0.7 at ~0.65 V_RHE_ | 0.1 M NH_3_ + 1.0 M KOH | *Nat Commun,* **2023**, *14*, 792 |
| 10 | NiCu-D-1:2/CP | Solvothermal | 44.9 at ~0.6 V_Hg/HgO_ | 0.055 M NH_4_Cl + 0.5 M NaOH | *ACS Appl. Nano Mater*. **2023**, *6*, 22, 20688–20699 |
